# Cultural landscape resilience evaluation of Great Wall Villages: A case study of three villages in Chicheng County

**DOI:** 10.1371/journal.pone.0298953

**Published:** 2024-04-18

**Authors:** Dan Xie, Meng Wang, Weiya Zhang

**Affiliations:** 1 School of Architecture and Art Design, Hebei University of Technology, Tianjin, China; 2 Shanghai Jiangong Sijian Group Co. Ltd., Shanghai, China; Hebei Agricultural University, CHINA

## Abstract

The Great Wall Villages (GWVs) are linked to the Great Wall in history, culture, and ecology. The cultural landscape resilience of Great Wall Villages (CLRGWVs) is distinctly significant. However, it is influenced by urbanization, pollution, and a lack of awareness of cultural landscape protection. Therefore, conservation and development practices still lack scientific strategies and guidance. This study proposes a new assessment system to quantify CLRGWVs, an analysis of the main influencing factors of resilience, and optimization paths to maintain sustainable development. Based on the socio-ecological system, this research designed the assessment with three criteria, eleven factors, and thirty-three indexes from the perspective of CLRGWVs. Furthermore, a demonstration test was constructed in Ningyuanbao Village, Dushikou Village, and Longmensuo Village in Chicheng County, Hebei Province, China. The results showed that there is some disparity between the three GWVs, with the resilience score of Dushikou Village being the highest in terms of resistance and learning. In contrast, Ningyuanbao Village’s resilience score is the lowest since resistance, recovery, and learning capacity are lower than in Dushikou and Longmensuo. Some influencing factors were found to be highly related to adaptive capacity. Lastly, some low-resilience aspects were identified as critical improvement targets for which corresponding optimization strategies should be proposed. This could be applied to streamline resilience optimization paths according to local conditions. This paper provides new ideas and directions for dealing with the sustainable development of villages and the conservation of cultural landscapes. It will also help villages deal with the relationship between socio-economic development and the conservation of cultural landscapes.

## 1. Introduction

### 1.1. Background

The Great Wall Villages develop from military settlements beyond the Great Wall, situated inside the 8,800-kilometer-long Ming Great Wall, from Hushan in Liaoning to Jiayu Pass in Gansu in the north of China. As the Ming Great Wall was mostly built on the ridges of high mountains in frontier areas, the Great Wall Villages were mostly located in remote areas of northern China, sparsely populated, economically backward, and with incomplete transport facilities. The GWVs have precious traditional resources and a unique cultural landscape, including not only landforms with military defensive characteristics but also important military cultural assets like elements of the border walls, beacon towers, and watch towers, as well as the defensive elements of the military fortresses, such as the walls, towers, forts, gates, and other cultural landscapes. Nowadays, these villages reflect the collisions and exchanges between agricultural civilizations and nomadic civilizations in ancient China and still play an important role in the inheritance of traditional culture. GWVs, transmitted from generation to generation, are constantly recreated by communities and groups in response to their environment and history. However, in the process of rural revitalization and urbanization, influenced by productive activities such as town building, agricultural reclamation, and the extraction of ore, GWVs are being destroyed and losing their cultural identity.

Most GWVs are in neither a protective area nor a restricted construction area. In addition, they are not cultural relic protection units. GWVs are far away from the Great Wall, but influenced by landform, the Wall and associated fortifications could still be seen from a large number of GWVs. In fact, they are part of the GWVs’ landscape. This paper takes unprotected GWVs with military cultural landscapes as the research object.

**Fig 1:**
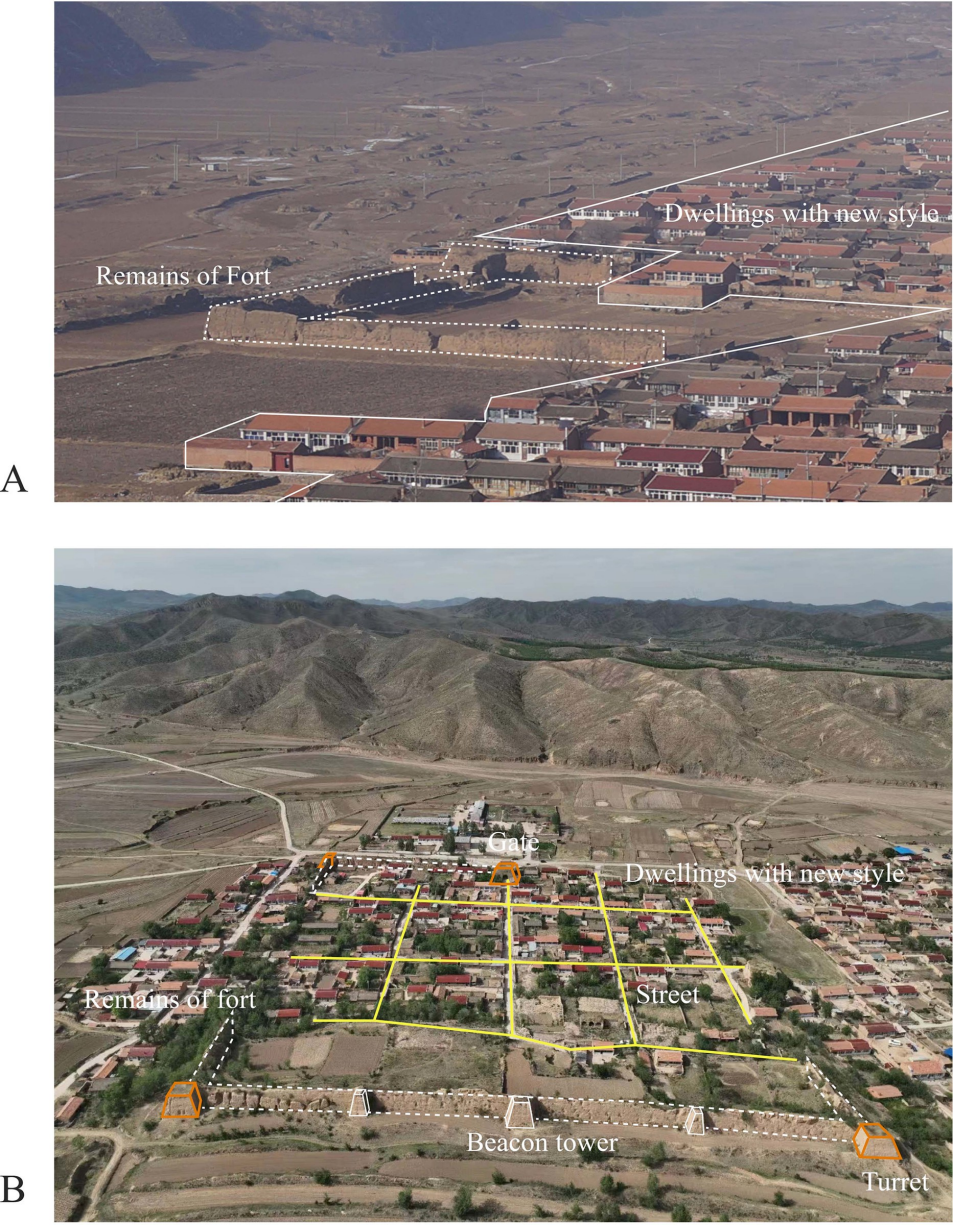
Remains of fort in GWVs. The base photo was provided by Xiaodong Ming.

Chicheng, a Great Wall Village-gathering area in the east of Hebei Province, is close to Beijing. In the Ming Dynasty, a large number of military settlements were densely distributed. However, the GWVs in Chicheng County have been seriously damaged in recent years, and a large number of tangible cultural landscapes have disappeared. The tangible cultural landscape of GWVs contain natural environment, defensive buildings, spatial form and signage landscape. In terms of the natural environment, Chicheng suffers from serious soil erosion due to natural disasters. In addition, affected by urbanization, the woodland in GWVs has been occupied. In terms of the defensive buildings, the fortified wall collapsed due to wind and water erosion. In addition, the Great Wall’s bricks were removed by locals to rebuild their homes, while fortress walls were plowed into ruin. The gates of most of the military forts have almost disappeared, and only part of the wall and the enemy platform remain. The internal defensive buildings in the fortress have disappeared, replaced by modern residential buildings. In terms of spatial form, the original cross-shaped and ding-shaped road patterns have been reserved, and some spatial patterns of forts have been replaced by a net-shaped pattern, signifying the growth in population and social progress. (Fig 1) In terms of the signage landscape, only some ancestral halls, temples, drum towers, and traditional theatrical buildings have been preserved. Therefore, the cultural landscape of GWVs needs to be urgently and scientifically protected by applying resilience theory in order to promote the sustainable development of GWVs.

(A) Status of Longmensuo Fort. Dotted line represents remains of fort, solid line represents dwellings with new styles. (B) Status of Songshu Fort. Dotted line represents remains of fort, yellow line represents roads in fort, orange line represents turret, white line represents beacon tower.Thus, this study introduces core concerns: under natural hazard risk and urbanization shock, the significance of the CLRGWVs is distinct, but the priority of conservation and development practices still lacks scientific evaluation. In this case, a specific resilience assessment system needs to be developed, and the main influencing factors of resilience and optimization paths need to be explored. The goal should be effective strategies for raising resilience to the inheritance pattern of the CLGWVs. In order to solve the problem that how to enhance the conservation of cultural landscape of Great Wall Villages for sustainable development, therefore, this study would build a resilience evaluation system for the cultural landscape of Great Wall Villages, to provide a quantitave method for measuring the resilience level. Then, this paper summarizes current issues in village resilience development and deeply explores optimization strategies to realize the conservation and utilization of the cultural landscape of Great Wall Villages.

#### 1.2. Literature review

With rapid urbanization and growing natural hazards, the cultural landscapes in villages are in danger and lack vitality since a significant number of physical and intangible rural cultural landscapes were not passed down from previous generations. A global agreement has been obtained on sustainable development. "The Hangzhou Declaration," signed in 2013, "Transforming Our World: The 2030 Agenda for Sustainable Development" [1], and "Sendai Framework 2015–2030 UNDRR" [2] have all expressed their continuous concern for sustainable development and explored resilient paths to handle disasters and adapt to social disturbances.

In China, renowned as a UNESCO World Heritage Site since 1987, the Great Wall is also an important Cultural Relic Protection Unit. In 2019, the Great Wall Conservation Master Plan was issued, defining the protection zone according to the principle "50 m on both sides of heritage as the protective area, 100m away from the protective zone in the city, and 500m in rural areas as restricted construction areas" [3]. Most of the GWVs are not Heritage Conservation Units because they are far away from the Great Wall, so their cultural landscape has not been taken seriously. Previous studies about village cultural landscapes have explored their definition, composition [4], evolutionary mechanisms [5], landscape characteristics [6], and conservation. In definition studies, the behavior of the people in the village is given more attention, and researchers study the cultural landscape from the perspectives of ecology, society, and culture [7, 8]. M. Cocks explored the concept of cultural landscape and suggested that a biocultural diversity perspective on heritage not only acknowledged the intrinsic connection between nature and culture but also elevated the importance of local people’s beliefs, values, and customs. In landscape characteristics studies, an evaluation system was constructed to explore the change of characteristics [9, 10]. Studies on the composition of military cultural landscapes [11], and evolutionary mechanisms of the settlement landscape [12], have become prevalent, and they provide a theoretical basis for conservation. In village cultural landscape preservation studies, researchers conducted quantitative analysis by building evaluation systems on the basis of classifying cultural landscapes, but they were restricted to static studies [13, 14].

For GWVs, many previous studies have covered different domains, including history, settlement systems, spatial layout, economic culture, construction techniques, and conservation. In the existing historical studies, the evolution of settlements [15], management systems [16], and ancient environmental factors about the siting of military forts [17, 18] have been increasing. In settlement system studies, researchers studied physical forms such as spatial layout [19], architectural types [20], structure of the system [21], and relationship with the Great Wall [22]. Among those studies about Great Wall Villages conservation, linear protection [23, 24], regional protection [25], individual village protection [26–28], constructing a value evaluation system to explore conservation strategies [29], and protection management mechanisms were paid more attention to. For example, Lin explored the spatial structure of intangible cultural heritage along the Ming Great Wall and constructed an appropriate development mode for the heritage corridors [30]. Zhang proposed the preservation of many fortifications according to their authentic historical sphere of control by clarifying the distribution of cultural heritage in Juyong Pass Defense Area [19]. While for GWVs, evaluation studies pay more attention to heritage value, different levels of static protection policies are developed depending on heritage value. There is a relatively small body of literature that is concerned with their cultural landscape. And only concern themselves with components of the military cultural landscape [31], architectural features [32], and evolutionary mechanisms. As a socio-ecological system, the CLGWVs are in a constant state of flux, characterized by dynamic evolution. Yet existing studies have not focused on the dynamic development of the cultural landscape and its sustainable development. Furthermore, the evaluation of the military cultural landscape has also not focused on resilience’s capacity to realize sustainable development. So resilience theory was employed to explore the dynamic resilience characteristics that adapt to challenges posed by natural hazards and man-made shocks.

Resilience is generally regarded as the ability of a system, community, or society exposed to shocks to resist, absorb, accommodate, and recover from disturbances, including through the preservation and restoration of its essential basic structures and functions [33, 34]. The notion of community resilience is gaining increasing significance in contemporary society, especially as many communities around the world appear to be gradually losing resilience in the face of challenges such as climate change, the outmigration of young people, and socio-economic ruptures [35, 36]. Previous research on community resilience has gradually shifted from the system’s recovery to its original state to the ability of transformation and learning, focusing more on influencing mechanisms [37–40], practical strategies [41–43], evolution and reconstruction [44, 45], building paths [46, 47], and resilience management [48]. Resilience evaluation is an important part of resilience research, and most of them establish evaluation index systems from the perspectives of economy, ecology, and society [49]. Some research focuses on resilience measurement from the perspective of resilience capacity, such as resistance, resilience, and other characteristics [50]. Assumma assessed the territorial resilience of a socio-ecological system through an innovative integrated evaluation framework to assist decision-makers and territory planners in the planning and management of resilient territorial systems [51]. Cristina analyzed how social-ecological resilience has been operationalized and measured and how systems react to several disturbances at the same time [49]. Giovanni defined an analytical tool—the rural diversity index—to assess the role of natural, economic, and social diversity in determining alternative rural socio-ecological development patterns [52]. However, this increasing interest in resilience-related themes to shocks has hitherto not generated studies investigating slow-onset disturbances affecting Great Wall Villages that are undergoing unprecedented natural and socio-economic change.

Together, these studies indicate that: (1) Previous studies on GVWs paid more attention to the structure of the defensive system, spatial layout, economic culture, and conservation; however, studies on CLGWVs lag behind. (2) Similar resilience analysis of villages is limited in system components, mechanisms, management, and characteristics, but there is still scant work on quantitative evaluation of the dynamic development process with resistance, restoration, and learning. (3) Research on the link between cultural landscape and resilience is still limited and fragmented.

### 1.3. Research aims

This study had two primary aims: (1) To make reasonable trade-offs between economic development, productive and living needs, and historical and cultural heritage conservation in order to solve practical problems. So we will introduce resilience theory into the assessment of the cultural landscape of the GWVs and then construct an interconnected framework between resilience and the socio-ecological system of the cultural landscape. To propose a new assessment system to quantify CLRGWVs and provide a basis for resilience measurement. (2) Guide the conservation of cultural landscapes and propose optimization paths based on the characteristics of GWVs so as to make them sustainable. By investigating those GWVs in Chicheng County and evaluating their current status in order to find existing issues and analyze resilience levels. According to different resilience levels to provide the corresponding optimum proposal.

Therefore, based on the socio-ecological system, an assessment framework of CLRGWVs from the perspective of resistance, recovery capability, and learning ability was constructed first. Secondly, this study chose Chicheng County in Zhangjiakou, a GWV-gathering area with hidden natural and social hazards, as an example. Using the case of three GWVs, this study analyzed the resilience of villages in the face of multiple and complex disturbances through a quantitative evaluation. Then, gray relational analysis (GRA) was used to analyze its main influencing factors, which were crucial to raising resilience. Finally, resilience optimization paths from three adaptive phases were proposed to achieve sustainable development of the cultural landscape.

## 2. Study area and data source

### 2.1. Study area

**Fig 2:**
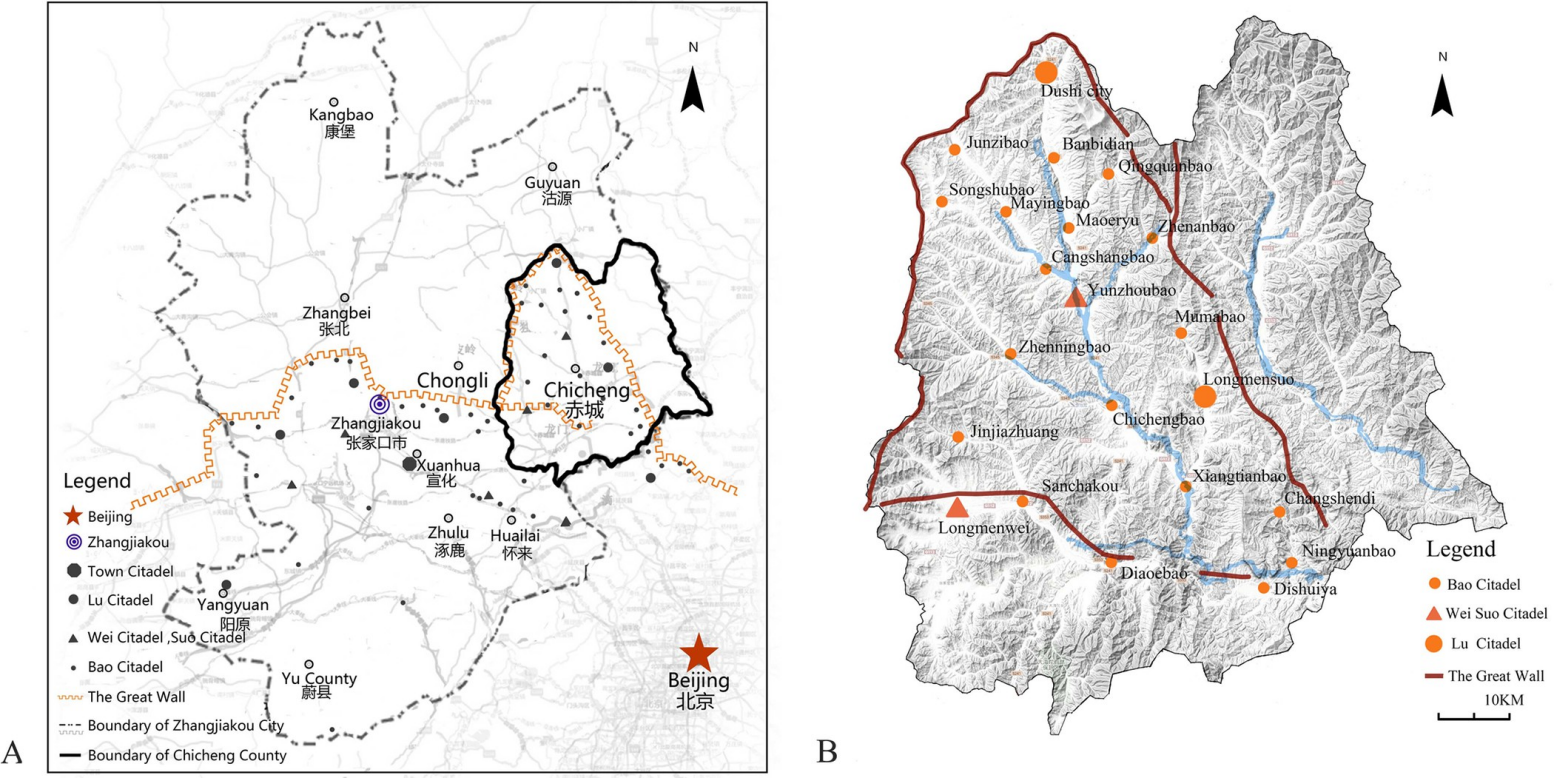
Distribution of GWVs. The base data was from USGS EROS: http://eros.usgs.gov/#.

**Fig 3:**
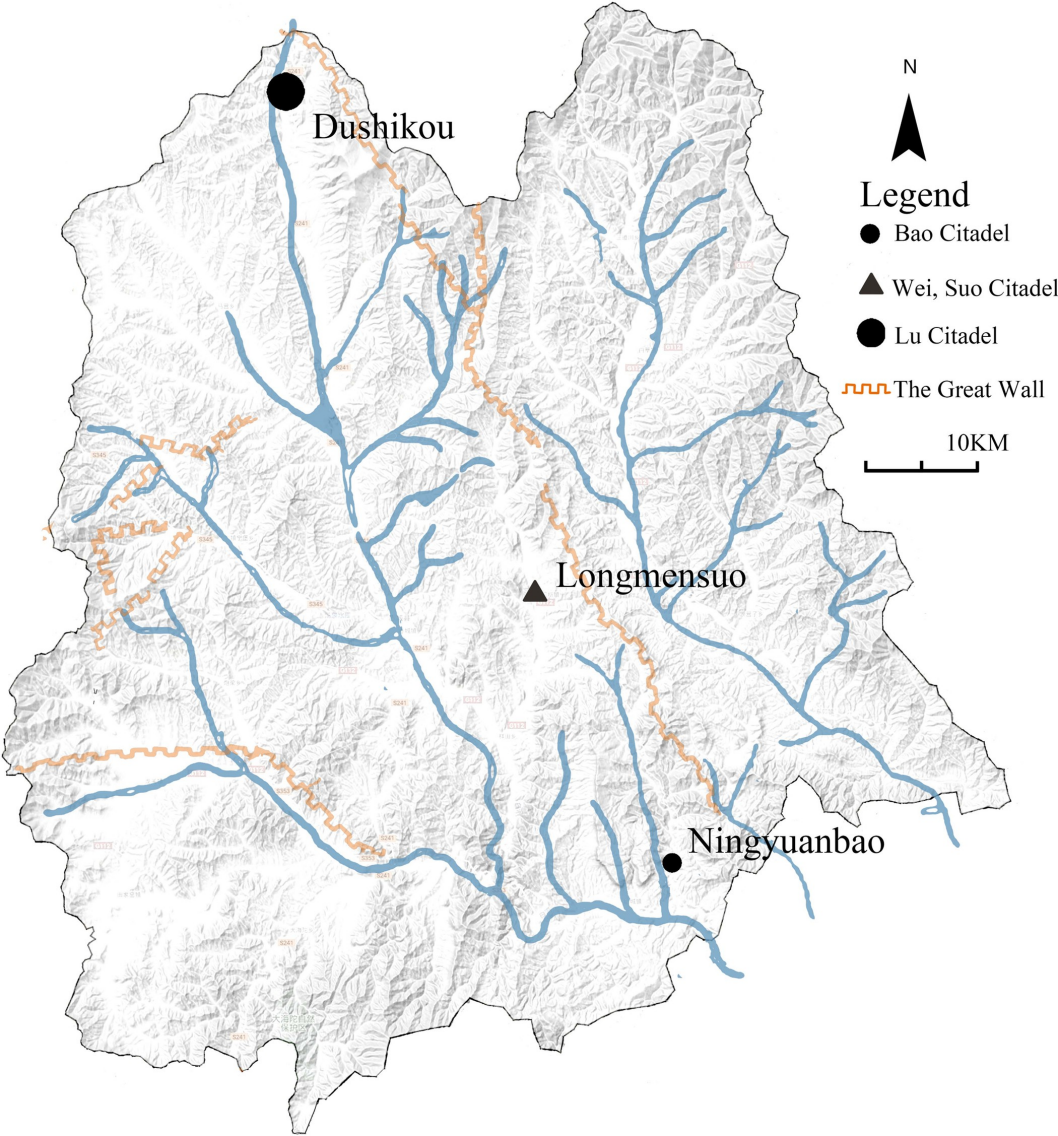
Distribution of surveyed villages. The base data was from USGS EROS: http://eros.usgs.gov/#. Small circle represents bao citadel, big circle represents lu citadel, triangle represents wei and suo citadel.

Located in the east of Zhangjiakou City in Hebei Province, China, Chicheng offers excellent research value and is typically used for the exploration of the CLRGWVs under environmental disaster risks and urbanization shock. It is situated in the overlap area between the Mongolian Plateau and the North China Plain. There are a large number of mountains and hills, with an average altitude of 945m. Chicheng County shows a slight slope from northwest to southeast, featuring a land climate in the form of an East Asian continental monsoon climate. Along the Ming Great Wall in Chicheng County, 22 military fortresses had been built, including beacon towers, watch towers, and so on. 279 kilometers of border walls are preserved today, with 87 watch towers, of which three are preserved completely, 12 are dilapidated, and only 72 have surviving bases or remnants [53] (Fig 2).

(A) Distribution of GWVs in Zhangjiakou. Red pentagram represents Beijing, blue circle represents Zhangjiakou. Black Octagon represents town citadel, black orbicular represents lu citadel, black triangle represents wei citadel and suo citadel, black dot represents bao citadel, orange line represents the Great Wall, the black dotted line represents boundary of zhangjiakou city, the black solid line represents boundary of chicheng county. (B) Distribution of GWVs in Chicheng County. Small circle represents bao citadel, big circle represents lu citadel, triangle represents wei and suo citadel, line represents the Great Wall. In the Ming dynasty, military settlements beyond the Great Wall were divided into five grades: town citadels, Lu citadels, Wei citadels, Suo citadels, and Bao citadels, on the basis of the military administrative system of Dusi Weisuo (Fig 3) The town citadel is the largest military and administrative center, with a circumference of over 7000m. Lu citadel is the command center of each Lu region, with a circumference of 2000–4000. Bao Citadel is the smallest unit of the military settlement system, with a perimeter of less than 2,000 meters.

The Great Wall settlements in Chicheng County were established one after another from Xuande to Wanli of the Ming Dynasty (1430–1588), and 22 Great Wall settlements such as Dushi, Yunzhou, and Chicheng were successively set up, with different military grades. Under the influence of regional environment, economy and culture, the military fortresses gradually evolved into today’s Great Wall Villages, which are divided into 15 administrative villages and 7 town administrative centers according to today’s administrative grade. In addition to the different grades, the Great Wall Villages in Chicheng County are also located in different geographic environments, and according to their spatial location, they are divided into three types, including 3 villages based on the ground, 11 villages based on the mountainous terrain, and 8 villages based on the plains. The economic level of the GWVs varies due to historical reasons, natural geographic conditions, and modern industries.

**Fig 4:**
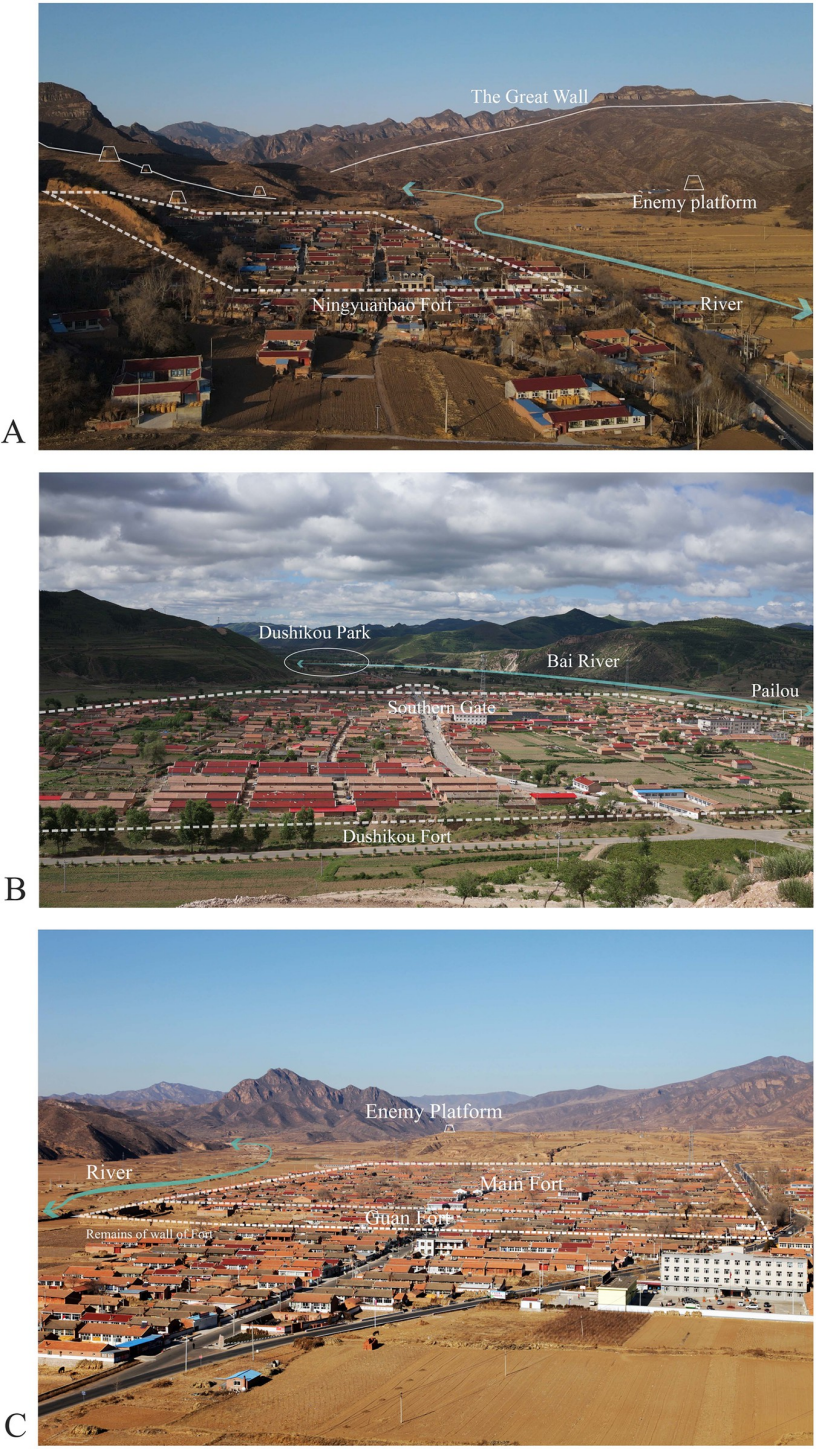
Status of Great Wall Villages. The base photo was provided by Xiaodong Ming. (A) Ningyuanbao Village. Dotted line represents fort, white solid line represents the Great Wall, trapezoidal represents enemy platform, blue line represents river. (B) Dushikou Village. Dotted line represents fort, blue line represents river, ellipse represents park. (C) Longmensuo Village. Dotted line represents fort, trapezoidal represents enemy platform, blue line represents river.

According to the principles of typicality of the case and feasibility and accuracy of the data obtained, based on the criteria of covering different grades and types (spatial pattern, economic level, and resident constitution), the research team selected three representative GWVs in Chicheng for the case study. Ningyuanbao is a Bao Citadel, and Longmensuo belongs to a type of Suo Citadel; both were set up for farming and training in the hinterland, while Dushikou is a Wei Citadel built for warning and battle, with an important military and economic position. Nowadays, there is a disparity in their current state of development. Ningyuanbao has now developed into the administrative village of Shangbao in Houcheng Town, which is a relatively backward economic development. Dushukou and Longmensuo are now developed as the administrative centers of the town, with good economic development but the biggest impact from urbanization. The three villages represent three different types in terms of spatial location selection, military grades in history, today’s administrative hierarchy, socio-economic levels, and so on. Therefore, these three GWVs could reflect the universal character of GWVs in Chicheng County. The basic information for three villages is shown in Fig 4 and S1 Table, S1–S3 Figs.

### 2.2. Data sources

The associated data sources utilized in this study include both statistical data and spatial data. Data from the government and an open online platform was combined with that obtained through field surveys, on-site surveys, a questionnaire, and a literature review. For statistical data, the population data was obtained from Chinese Census Information by Countryside, Township, and Street, Hebei Statistical Annuals, and the Hebei Government Services Website. Most historical information on the construction of fortresses comes from *Xuanfu Zhen Zhi*, *Chicheng County Zhi*, *Xuanhua Fu Zhi*, *and Jifu Tong Zhi*, a chronicle of the Ming Dynasty. Furthermore, the forest cover rate, land use rate, and other relevant data originated from the Chicheng County Government Website. For spatial data, the GWVs’ locations are based on site surveys and the Third National Heritage Census Report. Moreover, geographic information data and 30-m-leveled DEM data were obtained from a geographic space data cloud platform. Some quantitative indicators are acquired by ARCGIS 10.2 and Fragstata 3.4. These data sources were used in support of the resilience evaluation indexes and mainly influenced factors of resilience in this study.

## 3. Methods

A comprehensive evaluation system was constructed to understand how resilient a GWV is. In addition, resilience theory [54], with the method of AHP (Analytic Hierarchy Process), is an important theoretical foundation.The major steps were as follows: correlated framework construction, construction of the indicator system, preliminary screening of indicators, expert consultation, determination of weights, formulation of scoring standards, distribution of survey questionnaires, fuzzy comprehensive evaluation, correlation analysis of internal factors, etc. During the correlated framwork construction, applying resilience theory to the evaluation of cultural landscapes through qualitative research on two dimensions of system composition and characteristics. The method of AHP is employed to build an evaluation index system. Literature research and the expert scoring method are used to screen indices. The Delphi method is used to determine the relative importance of each indicator, and expert consultation is aimed at determining weights. During the process of data acquisition, spatial analysis by GIS, literature research, and survey questionnaires are employed to obtain comprehensive data. At last, the reliability analysis—Cronbach reliability analysis—is performed on CLREGWV’s score to estimate the internal consistency of the test. Grey Relational Analysis (GRA) was used to conduct a correlation analysis of internal factors.

### 3.1. Construction of the resilience evaluation system

The construction of the Resilience Evaluation System is based on resilience theory and the characteristics of resilient systems. A resilient system undergoes dynamic processes—before, during, and after perturbations—and makes adaptable changes in response to perturbation shocks. A similar study referenced is by Martin, who divided regional resilience into four dimensions: resistance, recovery, renewal, and reorientation [55].

Correlated framework construction is aimed at applying resilience theory to the assessment of the cultural landscape of the GWVs. This study associates the cultural landscape of GWVs with resilience systems in two dimensions: elemental composition and characteristics.

In terms of elemental composition, the cultural landscape is generally considered to be composed of two elements: culture and nature. Based on the classification and composition of traditional village cultural landscapes by scholars, this study proposes that the cultural landscape system of the GWVs is divided into two categories: tangible and intangible cultural landscapes [56]. Tangible cultural landscapes include natural environmental landscapes, fortified architectural landscapes, the spatial pattern of settlements, and public facilities. Intangible cultural landscapes include history, folklore, and production life.

The ecosystem generally contains elements such as the natural environment, architecture, and morphological patterns, and the social system generally contains elements such as history and culture, social structure, and values. The cultural landscape of the Great Wall Villages includes the natural environment, defensive buildings, spatial form, signage landscape, society and culture, production and life, and community relations. Therefore, the cultural landscape of the Great Wall Villages is both ecological and social, so we take the socio-ecological system as the influencing factor of the Great Wall Villages.

According to the theory of socio-ecological systems, cultural landscape resilience systems are composed of ecosystems and social systems. The study correlates the tangible and intangible cultural landscapes with the ecosystem and social system of the resilient system, respectively. Ecological resilience refers to the ability of an ecosystem to withstand and recover to sustain itself without fundamental changes to the structure of the system when subjected to external disturbances. Species diversity and landscape pattern diversity are important expressions of ecological resilience. For GWVs, the natural environment is the natural base of the village; the fortified architectural landscape and the spatial pattern of the village provide the cultural context, which is an important part of the CLGWVs. All tangible cultural landscapes form the ecosystems in the cultural landscape resilience system of Great Wall Villages. Social resilience is the ability of social systems to recover in the face of external disturbances, mainly in the socio-cultural sphere, more reinforcing group or organizational relationships and behavior, and historical and cultural contexts. For GWVs, social resilience is the ability for intangible landscape elements to resist disturbances of social, environmental, and human nature. Traditional cultures, such as folk culture, religious beliefs, and military thought, are historical and cultural resources for GWVs, providing a cultural basis for the diversity of productive village life. The group organization and social structure of a village determine the security and stability of social systems. All intangible cultural landscapes form the social systems in the cultural landscape resilience system of Great Wall Villages.

In terms of characteristic association, the cultural landscape of GWVs has a variety of characteristics such as wholeness, diversity, and dynamism. The resilience system has the characteristics of the resilience stages of resistance (resistance stage), recovery (recovery stage), and learning (learning stage), and the study correlates them with the cultural landscape system’s features of integrity, diversity, and dynamism. Resistance indicates the ability of a system to resist and buffer against external disturbances. In the cultural landscape of the GWVs, the resistance characteristic requires the preservation of the integrity and richness of the cultural landscape resources. Recovery means that the system is able to adapt and adjust to the impact of disturbances, taking a variety of response measures to restore the system to its original state. In the cultural landscape of GWVs, the recovery feature emphasizes the ability of villages to eliminate the adverse impacts of external disturbances, such as landscape destruction, architectural destruction, loss of functions, disappearance of traditional culture, etc. In this process, the system needs to pay attention to the diversity of landscape patterns and functions, as well as the protection and inheritance of cultural heritage, so as to achieve the perfection of cultural landscape resources. Learning capacity refers to the ability of the cultural landscape to be renewed or reorganized after the shock, enabling the cultural landscape system to have the capacity to transform and innovate and thus achieve a better state of stability. In the cultural landscape of GWVs, learning capacity emphasizes the inheritance of historical and cultural resources and the integration of social values into the development of the area so that the cultural landscape can be developed sustainably.

**Fig 5:**
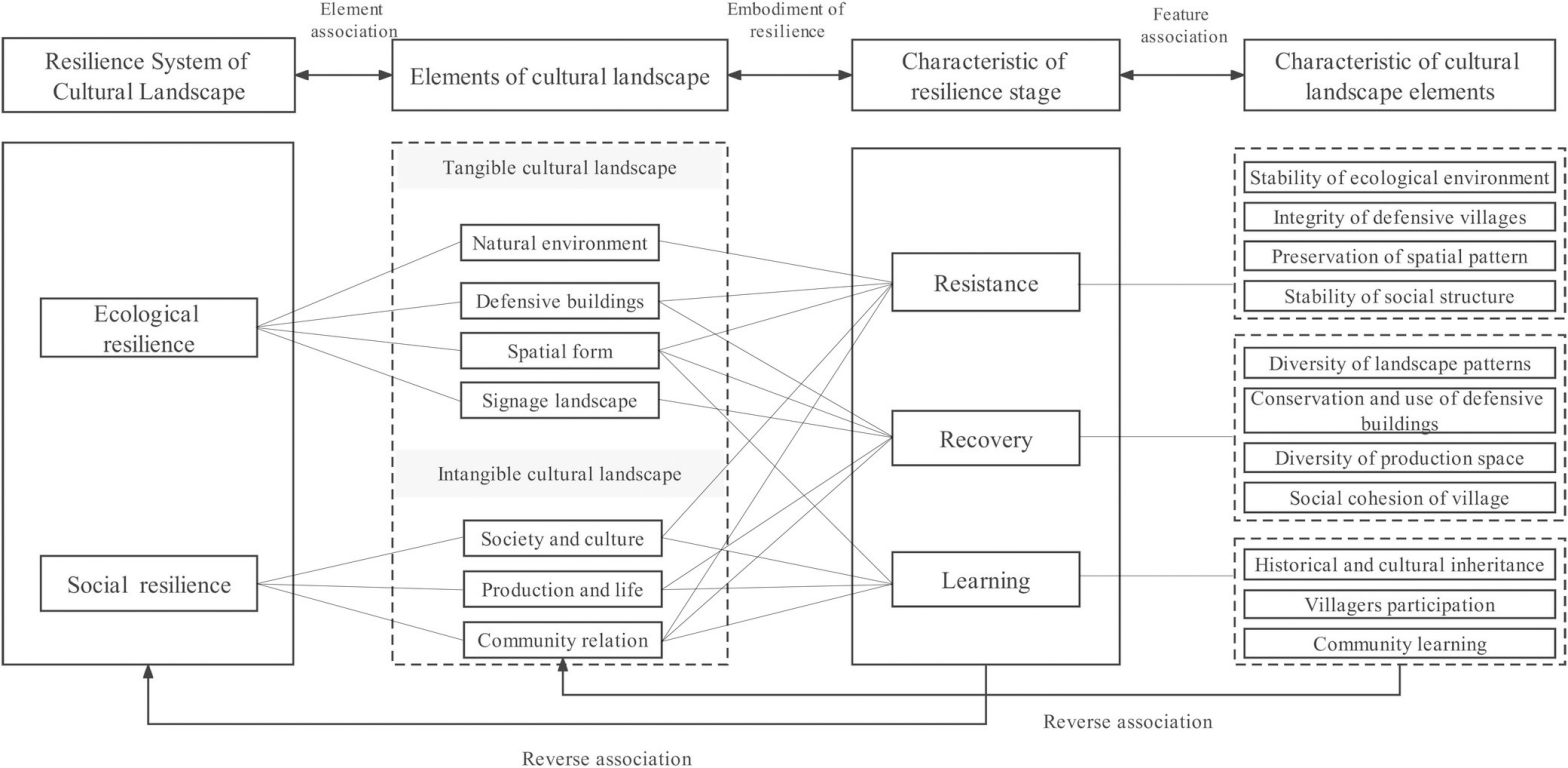
The framework of CLGWVs and resilience theory.

**Fig 6:**
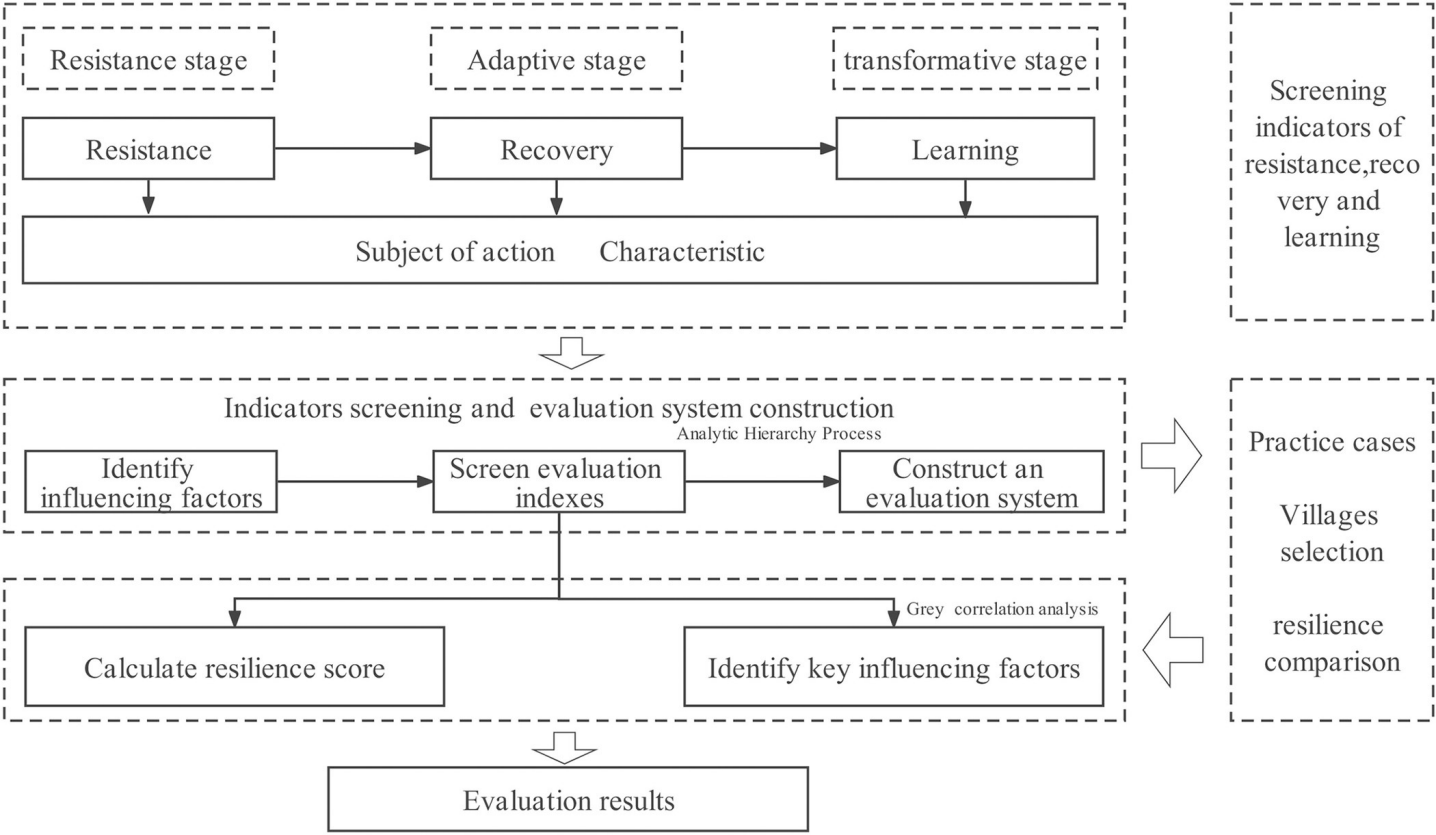
Path for construction of resilience evaluation system.

Lastly, the relevance framework between resilience theory and cultural landscape is established (Fig 5). The evaluation process is shown in Fig 6 below.

### 3.2. Selection of the resilience indexes

The Analytic Hierarchy Process (AHP) was used to construct the evaluation system for the CLGWVs. The AHP method is suitable for dealing with complex systems related to make a choice from several alternatives and which provides a comparison of the considered options. The AHP enables the Decision Maker to structure a complex problem in the form of a simple hierarchy and to evaluate a large number of quantitative and qualitative factors in a systematic manner with conflicting multiple criteria [57]. In AHP, preferences between alternatives are determined by making pairwise comparisons, in which the DM examines two alternatives by considering one criterion and indicates a preference. These comparisons are made using a preference scale, which assigns numerical values to different levels of preference [58].

**Fig 7:**
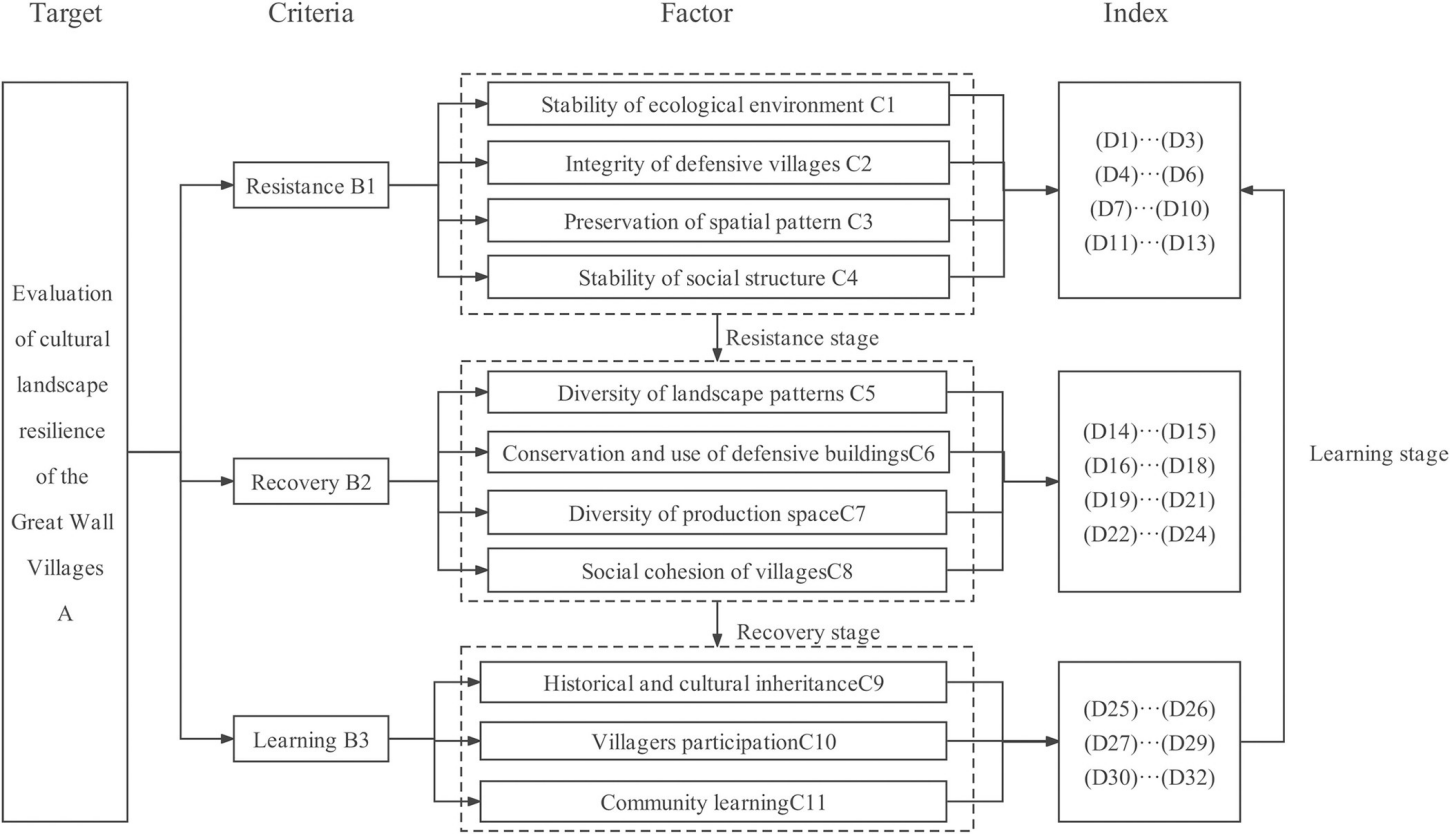
Framework of resilience evaluation system.

The evaluation system contains four levels: target layer A, criteria layer B, factor layer C, and indicator layer D. Based on a correlation framework, considering resilience’s stage characteristics, the indicators of resistance, recovery, and learning were selected. The choice of indexes should combine resilience theory with requirements for *the protection and development of cultural landscapes*, *as well as taking into account the relevant regulations for the protection and development of traditional villages*, *the evaluation and recognition system of traditional villages*, *the protection of famous historical and cultural cities*, *towns*, *and villages*, and other normative standards related to cultural landscapes. Moreover, in accordance with the principles of orientation, scientificity, authenticity, and feasibility, this system referred to many other materials, such as the afore-mentioned literature, as well as the opinions of experts and GWV’s managers. Furthermore, the feasibility of the index system for collecting data in underdeveloped regions was fully considered. The assessment system consisted of 3 criteria, 11 factors, and 32 indexes (Fig 7).

In terms of index choice, the literature was studied in terms of both rural resilience assessment and cultural landscape assessment studies, and appropriate indicators were selected. Firstly, in the study of resilience evaluation indicators, the indicators that characterize both ecological and social resilience are collated. Also, the evaluation of traditional village landscapes from the perspective of vulnerability has some reference to this paper [59, 60]. Secondly, evaluation indicators related to the cultural landscape of the Great Wall villages were collected. A literature review of evaluation systems related to rural village landscapes [61, 62], evaluation of landscape heritage in traditional villages [63, 64], evaluation of cultural landscape conservation [65], and research on the value evaluation of the Great Wall defense system [66] was done so as to screen out suitable resilience evaluation indicators for the CLGWVs. Lastly, indicators reflecting the ecological and social resilience of the CLGWVs were identified.

#### 3.2.1. Resistance B1

The resistance of the cultural landscape to shock by urbanization and natural disasters is the ability to absorb and buffer changes according to its own resources, which are composed of stability of the ecological environment (C1), integrity of defensive villages (C2), preservation of spatial pattern (C3), and stability of social structure (C4). The stability of the ecological environment in the cultural landscape is conducive to the construction of ecological resilience. Based on relevant studies, the vegetation cover (D1), landscape patch density (D2), and topography (D3) are selected to measure the stability of the natural ecological environment. Maintaining the integrity of the defensive landscape of the GWVs is a basic requirement for cultural landscape conservation. According to the research related to the conservation evaluation of the Great Wall defensive system, the deterioration degree of the remains (D4), longevity of history (D5), and military grade (D6) were selected to represent its historical and cultural value.

Specifically, spatial pattern preservation consists of the characteristics of site suitability (D7), integrity of village morphology (D8), completeness of public facilities (D9), and road accessibility (D10); the higher the scores of D7, D8, D9, and D10, the better the spatial pattern preservation and the more stable the overall cultural landscape structure. According to the former evaluation system for the protection of historical and cultural villages and towns, the degree of folklore maintenance (D11) was selected to reflect social and cultural stability. Population and social composition are the main factors in evaluating the stability of the social structure of an area; hence, the proportion of indigenous people in the total population (D12) and the proportion of people leaving villages (D13) are selected to reflect the stability of the villages’ social structure.

#### 3.2.2. Recovery B2

The recovery characteristic of the CLGWVs is measured by functional diversity of resources, composed of diversity of landscape patterns (C5), conservation and use of defensive buildings (C6), diversity of production space (C7), and social cohesion of the village (C8). As for the layer of diversity of landscape patterns (C5), which is from the concept of landscape heterogeneity put forward in landscape ecology, the higher the scores of such landscape diversity indexes (D14) and landscape dominance indexes (D15), the better the natural environment of the village, the stronger its recovery, and the more conducive it is to the cultural landscape protection of the village. Meanwhile, the conservation and development status of defensive buildings (C6) is crucial for the recovery of the cultural landscape, so it is necessary to pay more attention to the Great Wall, beacon towers, watch towers, and village gates, especially the protection level of defensive buildings (D16), the grade of villages (D17), and the historic building renovation rate (D18). The diversity of production space C7 can roughly reflect the adaptability and resilience of the village cultural landscape. A higher spatial richness of the village (D19), regional recognition of the street landscape (D20), and agricultural landscape types (D21) mean better cultural landscape recovery. For social resilience, villagers’ sense of belonging (D22) and political participation (D23) can reflect the village’s social cohesion, while labor force ratio (D24) reflects the resilience of the social structure.

#### 3.2.3. Learning B3

Learning ability is featured with the transformation and innovation ability of historical and cultural resources, including historical and cultural inheritance (C9), villager participation (C10), and community learning (C11). The higher the scores of attractiveness of folklore activities (D25) and richness of intangible culture (D26), the more attractive it is and the better the inheritance of culture. Community organizations include two major parts: villagers and the government. From the villagers’ perspective, the awareness of cultural heritage protection (D27) and heritage acceptance (D28) can reflect the villagers’ learning ability of the cultural landscape; meanwhile, their self-directed learning capacity reflects their ability to adapt to external disturbances, as reflected by the proportion of educated population (D29). For government, community learning (C11) is composed of relevant policy mechanisms (D30) and conservation, development planning (D31), and the extent of tourism development (D32).

### 3.3. Data processing and resilience calculation

#### 3.3.1. Weight determination

The weights are determined by the classical Analytic Hierarchy Process (AHP). First, a tree hierarchical structure is constructed according to the resilience evaluation framework of CLGWVs, and then the score questionnaire is distributed to experts and scholars by Yaahp software [67]. The experts were from four domains: the field of the Great Wall’s defensive system, the Great Wall’s heritage protection, traditional village protection, and settlement landscape. They came from the cities of Tianjin, Beijing, and the province of Hebei, familiar with the GWVs. Two experts in each field were invited to score according to pictures and basic data sent by email in May 2022. Based on scoring criteria for factor scale importance 1–9 (S2 Table), the experts gave the corresponding weight scores. Then, the software will generate a judgment matrix to obtain the weight of each index in the resilience assessment index system (S2 and S3 Tables). Then, in order to avoid the occurrence of contradictory judgment results, the results are tested for consistency, and the data are imported into yaanp software for calculation, and when the consistency ratio CR < 0.1, it is considered to pass the consistency test. Finally, after normalizing the above calculation results, the weight value of each level indicator is obtained.

#### 3.3.2. Determination of scoring standards and survey questionnaire design

For qualitative indicators, the graded scoring method is used to determine each indicator’s scores. There are 5 grades of scores, and each grade is assigned 1 point, which is in the interval of 1–1, 2–2, 3–3,4–4,5–5, respectively [36]. For some quantitative indicators like vegetation cover D1, landscape patch density D2, and landscape diversity index D14, the data is from the analysis of land-use data by ARCGIS 10.2 and Fragstata 3.4, and a five-grade centesimal system similar to the above method is developed in combination with relevant standards for scoring. For questionnaire and interview indicators, the data were from field surveys and questionnaire surveys. The majority opinion results of the questionnaire interview are the final results. The survey questionnaires were distributed to interviewees, which were composed of four parts: experts in the field of the Great Wall defensive system, the Great Wall heritage protection, traditional village protection, and settlement landscape; representatives of villagers; managers of village-related administrative organizations; and tourists. In the questionnaire, the problems Great Wall Village was confronted with and the suggestions for resilience optimization were included. From this, we could know people’s views on conserving the ecology and culture of the area. In terms of representatives of villagers, more than 50 questionnaires were guaranteed to have been collected in each village. This sample data has been maximized due to the fact that the Great Wall Villages are generally heavily hollowed out, with the vast majority of adults going out to work, leaving behind the elderly and some children. Furthermore, more than 60 questionnaires were guaranteed to have been collected from experts, more than 100 questionnaires from managers of village-related administrative organizations and more than 100 questionnaires from tourists.

#### 3.3.3. Resilience calculation

Lastly, the weights of the indicators were multiplied by the actual scores of the indicators. Finally, the results of all indicators were summed up to obtain the overall resilience score. The higher the score, the higher the resilience level. Specifically, this paper disassembles the resilience process: resistance, recovery, and learning all represent the resilience of the system in the process area under perturbation. They are all positive indicators, so the process resilience level and the resilience composite index are formulated as follows:

$$X=\sum_{i=1}^{13}WiPi,(i=1,2,\ldots n)$$ (1)

$$Y=\sum_{i=14}^{24}WiPi,(i=1,2,\ldots n)$$ (2)

$$Z=\sum_{i=25}^{32}WiPi,(i=1,2,\ldots n)$$ (3)

R=X+Y+Z (4)

X, Y, and Z represent the resistance, resilience, and learning indices, respectively. Wi represents the indicator weight value, Pi represents the actual value of indicator I, and R is the resilience composite index.

#### 3.3.4. Reliability analysis

The formula of the reliability analysis—Cronbach reliability analysis—is as follows:

$$\alpha =\frac{K}{K-1}\left(1-\frac{\sum S_{i}^{2}}{S_{x}^{2}}\right)$$ (5)

*α* is the reliability coefficient, *K* is the number of test items, $S_{i}^{2}$ is the score variation of all subjects on the *i*-th question, and $S_{x}^{2}$ is the variance of the total scores obtained by all subjects. If the reliability coefficient is less than 0.35, the scale data is unreliable. A reliability coefficient larger than 0.8 is acceptable. If it is larger than 0.9, the scale data is of high reliability.

### 3.4. Grey relational analysis

A gray relational analysis was suggested by Deng (1989) [68]. The GRA is a technique for determining how closely the sequences approximate one another. The basic idea is to determine whether a system is closely linked by looking at the geometric similarity of the reference and comparison series. To generate an overall comparison of the choices, GRA has four processes. 1 preparation of factor compatibility; 2 derivation of reference sequences; 3 calculation of gray relational coefficient; 4 determination of gray relational grade (56). In this study, in order to compare the association of each index with the level of resilience, the reference sequences are the results of resilience levels R1, R2, and R3, and the comparison sequences are the indices. Then, calculate the correlation coefficients between each comparison sequence and the corresponding element of the reference series separately. The formula is shown in (6). Lastly, the average of the correlation coefficients at each moment is used as the final correlation; the formula is shown in (7). The greater the correlation, the closer the development of the comparative series is to the reference series, and the indicator is more closely related to the level of resilience.


$$\zeta_{i}(j)=\frac{{min}_{i}{min}_{j}|R_{i}-X_{i}^{\prime }(j)|+\rho *{max}_{i}{max}_{j}|R_{i}-X_{i}^{\prime }(j)|}{\left|R_{i}-X_{i}^{\prime }(j)|+\rho *{max}_{i}{max}_{j}|R_{i}-X_{i}^{\prime }(j)\right|}(i=1,2\ldots n;j=1,2\ldots m)$$ (6)

In the formula, *ρ*∈(0,∞), *ρ* = 0.5.


$$r_{j}=\frac{1}{n}\times W_{j}\times \sum_{i=1}^{n}\zeta_{i}(j)(i=1,2\ldots n;j=1,2\ldots m)$$ (7)

Key indices have a greater influence on the resilience of the cultural landscape of GWVs. By using gray relationship analysis, the key indices can be identified, which will provide data to support and inform their resilience optimization strategy.

## 4. Results

### 4.1. Comprehensive resilience evaluation results

According to the resilience evaluation index system constructed above and questionnaire survey (S4 Table), the scores are shown in S5 Table. In this table, village names are abbreviated, such as Ningyuanbao Village (NYB), Dushikou Village (DSK), and Longmensuo Village (LMS). Then, reliability analysis is conducted, and the result shows that the average value of the alpha index reaches 0.931 and the alpha index of each indicator is above 0.92, showing that the scale has high consistency and reliability.

**Fig 8:**
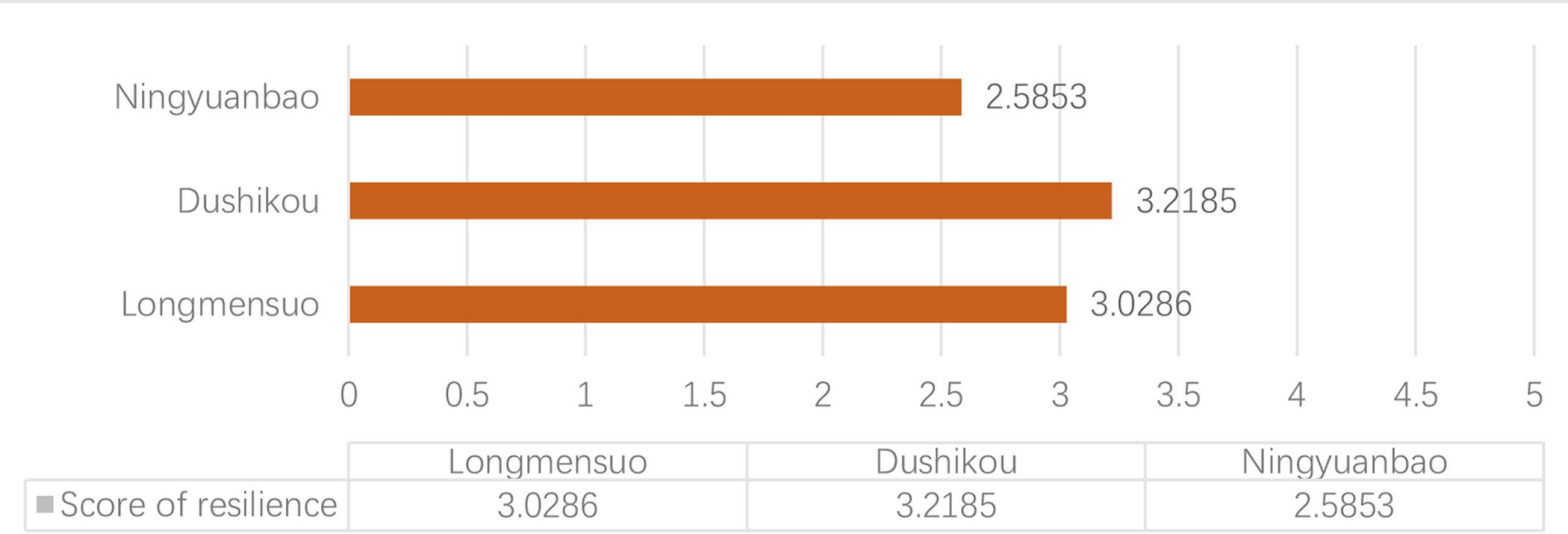
General evaluation results of the CLREGWVs.

After the calculation of resilience, we could see from Fig 8 that Dushikou Village has the highest score (3.2185), followed by Longmensuo (3.0286) and Ningyuanbao Village (2.5853 points), ranking 2nd and 3rd, respectively. Dushikou Village has the most resilient cultural landscape, while Ningyuanbao has the worst ability to resist and recover from disturbances. For Dushikou Village, the Great Wall here is well preserved, and it has been named a national cultural heritage protection unit. As a result, Dushikou Village has received better policy support, which has led to greater maintenance of the cultural landscape and a higher level of community learning. Ningyuan Bao Village is geographically remote, which affects the economic development of the whole village. Additionally, villagers lack participation and a sense of belonging in village development, so the whole resilience level is lowest.

**Fig 9:**
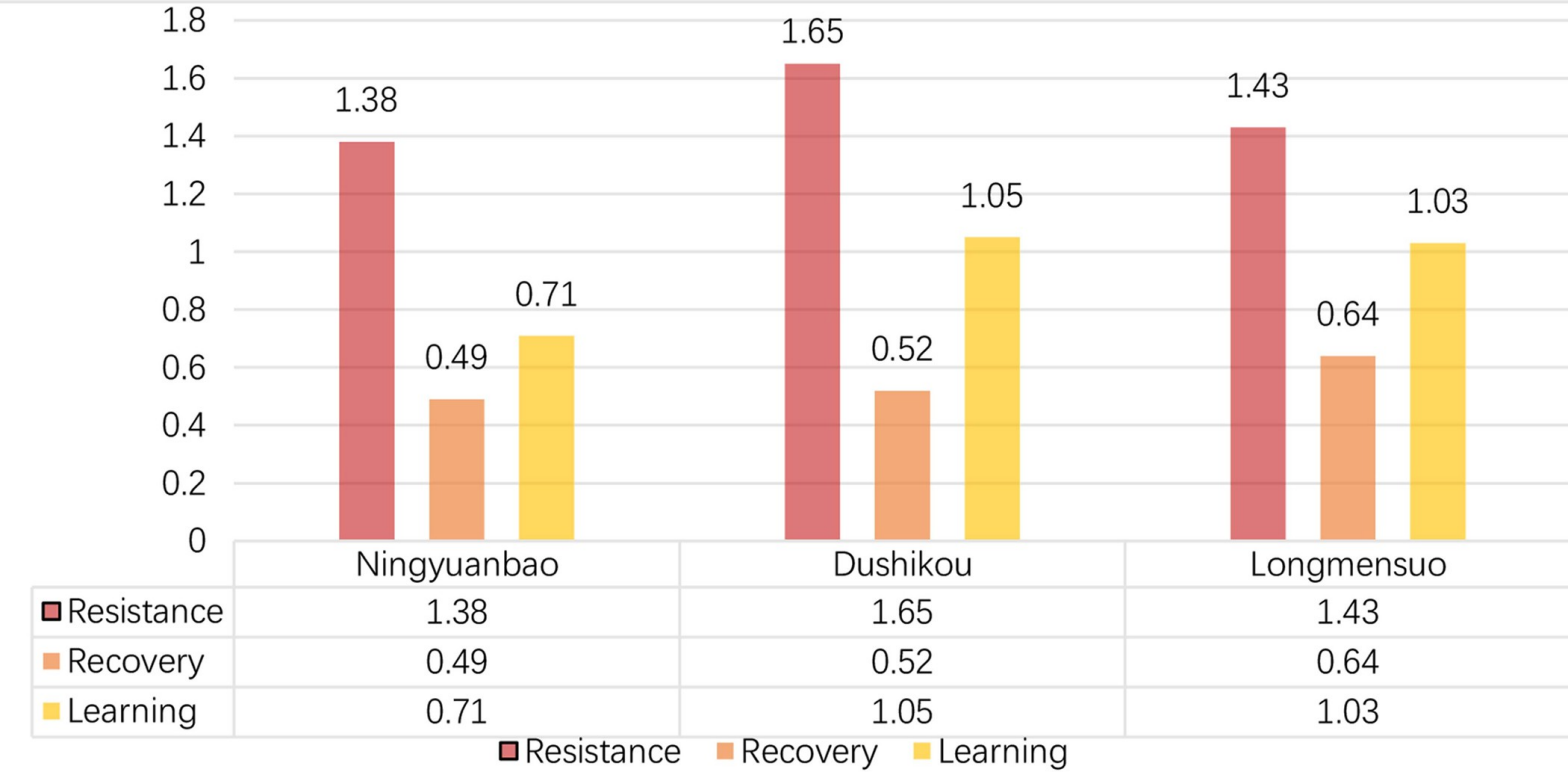
Staged evaluation results of the CLREGWVs. Red represents resistance, orange represents recovery, yellow represents learning.

According to Fig 9, Dushikou Village has the highest values in terms of resistance and learning capacity, indicating that it has an obvious advantage in terms of stability of the ecological environment, integrity of defensive villages, preservation of spatial patterns, and stability of social structure. Additionally, benefiting from tourism development and policy support, Dushikou has a strong ability to adapt to shocks. Disrupted spatial layout and low levels of community organization lead to Ningyuanbao’s low values in resistance and learning, respectively. In terms of recovery, three villages have similar values at a balanced level of resource adjustment. Furthermore, three villages show the same phenomenon of resistance being highest, followed by learning and recovering, ranking 2nd and 3rd, respectively.

### 4.2. Factor layer resilience evaluation results

In addition to the general overview of the three GWVs’ resilience, it is necessary to find the weaknesses and the parts that most affect the resilience of the cultural landscape, so a comparison of the factor indicators across the villages is required.

**Fig 10:**
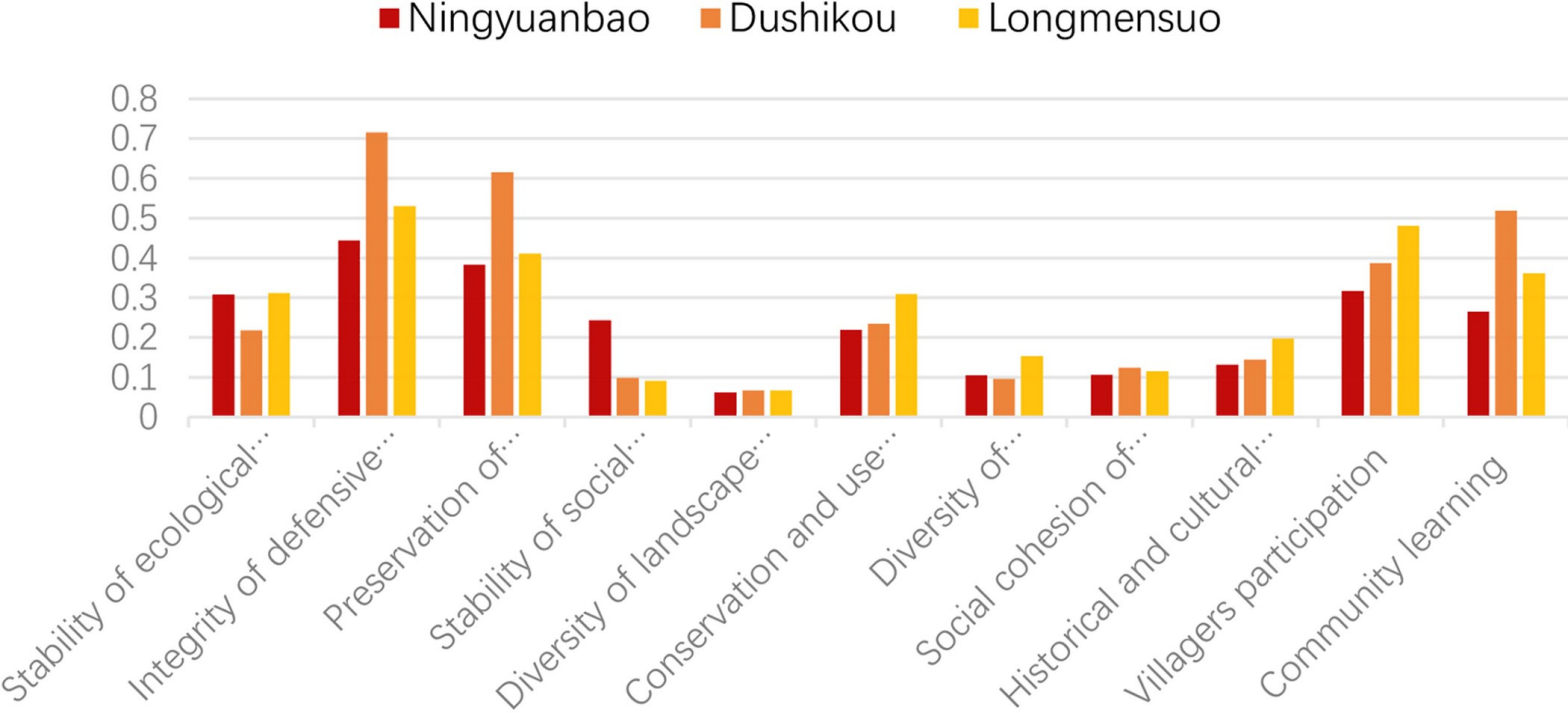
Comparative evaluation results of three villages. Red represents Ningyuanbao, orange represents Dushikou, yellow represents Longmensuo.

According to Fig 10, all three villages perform best in terms of integrity of defensive village (C2) and preservation of spatial pattern (C3), indicating that elements and patterns of cultural landscapes in the three GWVs are well protected. As the least resilient village, Ningyuanbao has the highest value only in terms of the stability of its ecological environment among the three villages because it is far away from the economic center and receives less attention, especially in the context of rapid modernization. Therefore, the ecology of the village has not been damaged by modern construction. In contrast, the most resilient Dushikou Village has the lowest level of ecological spatial stability. Among the indicators related to recovery, Longmensuo has an advantage in C6 and C7, which indicates that the diverse spatial functions, flexible street layout, and high utilization of traditional buildings are favorable to raising the resilience of the cultural landscape of the villages. In the learning dimension, villager participation and community learning perform relatively well, with both Dushikou and Longmensuo getting higher scores. This suggests that policy assurance and planning interventions are conducive to improving the ability to cope with disturbances.

For Ningyuanbao Village, the scores of integrity of defensive villages (C2) and preservation of spatial pattern (C3) are higher, at 0.4439 and 0.3828, respectively. Less affected by urbanization, the fortified buildings and natural environment were preserved better, which promoted the transmission of cultural diversity. Strong cultural stability makes villages more resistant to natural hazards and social disturbances. However, the scores of landscape pattern diversity (C5), production and living space diversity (C7), and social cohesion of the village (C8) are relatively low: 0.0623, 0.1409, and 0.1067, respectively. Ningyuanbao Village’s low landscape pattern index leads to a low restoration capacity for ecological space. In addition, the low diversity of productive and living landscapes affects biodiversity, so the recovery of ecological space is poor. Furthermore, far away from regional economic centers and lacking adequate infrastructure, there is no mainstay industry in the village; therefore, some residents have moved out to find a job, and more and more buildings are vacant, leading to derelict cultural heritage and a low level of social cohesion.

For Dushikou Village, integrity of defensive villages (C2), preservation of spatial pattern (C3), and community learning (C11) get higher values, 0.7155, 0.6149, and 0.519, respectively. In terms of defensive landscape, the Great Wall in the Ming Dynasty is preserved well, and most of it is built with stones. The fortress is a protected cultural heritage unit in Hebei Province, and most of its walls are preserved, which has become a major tourist resource for the village while promoting the preservation of the cultural landscape and development. In spatial pattern, Dushukou Village has the natural defensive barrier of the natural environment. It is situated in a hinterland bordered by mountains to the north, west, and east. The Bai River runs through the west side of the fortress. So that village still retains its traditional spatial pattern and has stable security of material resources to ensure self-sufficient energy for village livelihoods. All of these are conducive to improving the resistance capacity of resilience. However, the scores of social structure stability (C4), diversity of landscape pattern (C5), and diversity of productive and living space (C7) are low. Dushikou’s landscape diversity index (D14) and dominance index are low, reflecting its poor ability to restore ecological space. Being close to the economic center and influenced by the involvement of tourism development companies, the ecological space taken up by modern construction. In addition, its lower vegetation cover, higher patch density, and the ecological space taken up by construction result in low ecological stability.

For Longmensuo Village, the scores for integrity of defensive village (C2), preservation of spatial pattern (C3), and participation of villagers (C10) are high, 0.5308, 0.4105, and 0.4914, respectively, while the score for diversity of landscape patterns is the lowest. The villagers’ participation (C10) shows that the local government has adopted a variety of policies and activities to build villages, and the inhabitants of villages have a good awareness of heritage conservation and a sense of local identity, which is conducive to policy implementation and project execution. Eventually, the village can realize sustainable development. In addition, Longmensuo is distributed along the river, with a large area for construction and a high degree of fragmentation, which is not conducive to the development of a diverse and dominant landscape pattern and ecological resilience. The low scores for stability of social structure (C4) and social cohesion of the village (C8) reflect the village’s disadvantages in socio-cultural and demographic structure; the score of 0.309 for conservation and utilization of defensive buildings (C6) represents a good reconservation and re-utilization of historical buildings, which is beneficial to the restoration of the cultural landscape.

### 4.3. Identification of key indicators for resilience

Then, according to the calculated results of resilience, GRA was used to analyze the mainly influencing factors of resilience. GRA is a part of grey system theory which is appropriate for solving issues involving complex interactions between several components and variables [69].

#### 4.3.1. Key influencing factors of resistance

**Fig 11:**
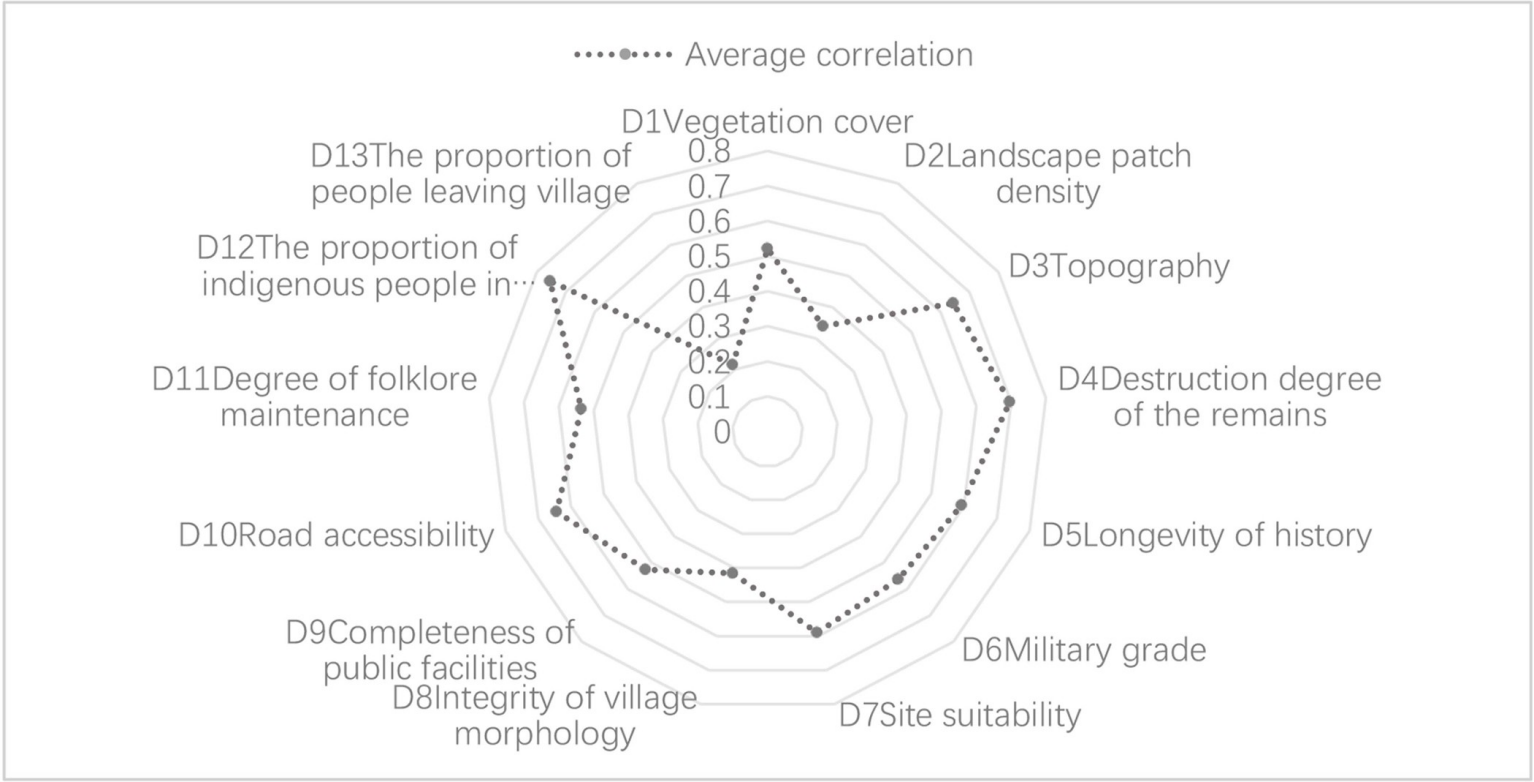
Correlation analysis of resistance of GWVs in Chicheng County.

In the resistance dimension, as can be seen in Fig 11, the correlation of topography (D3), deterioration degree of the remains (D4), road accessibility (D10), and the proportion of indigenous people in the population (D12) is high, indicating that these indicators have the greatest influence on the resistance of the GWVs and are the most significant factors in the resistance indicators. D12 is most related to resistance capacity because indigenous inhabitants are an important support for villages’ sustainable development. The trend towards hollowing out villages is increasing as more and more indigenous people go to the city to work, which will lead to a lack of vitality in the development of the villages and the loss of some of the fortified landscapes. In contrast, the proportion of people living outside the village (D13) and the density of landscape patches (D2) have a lower influence on the overall resilience level.

#### 4.3.2. Key influencing factors of recovery

**Fig 12:**
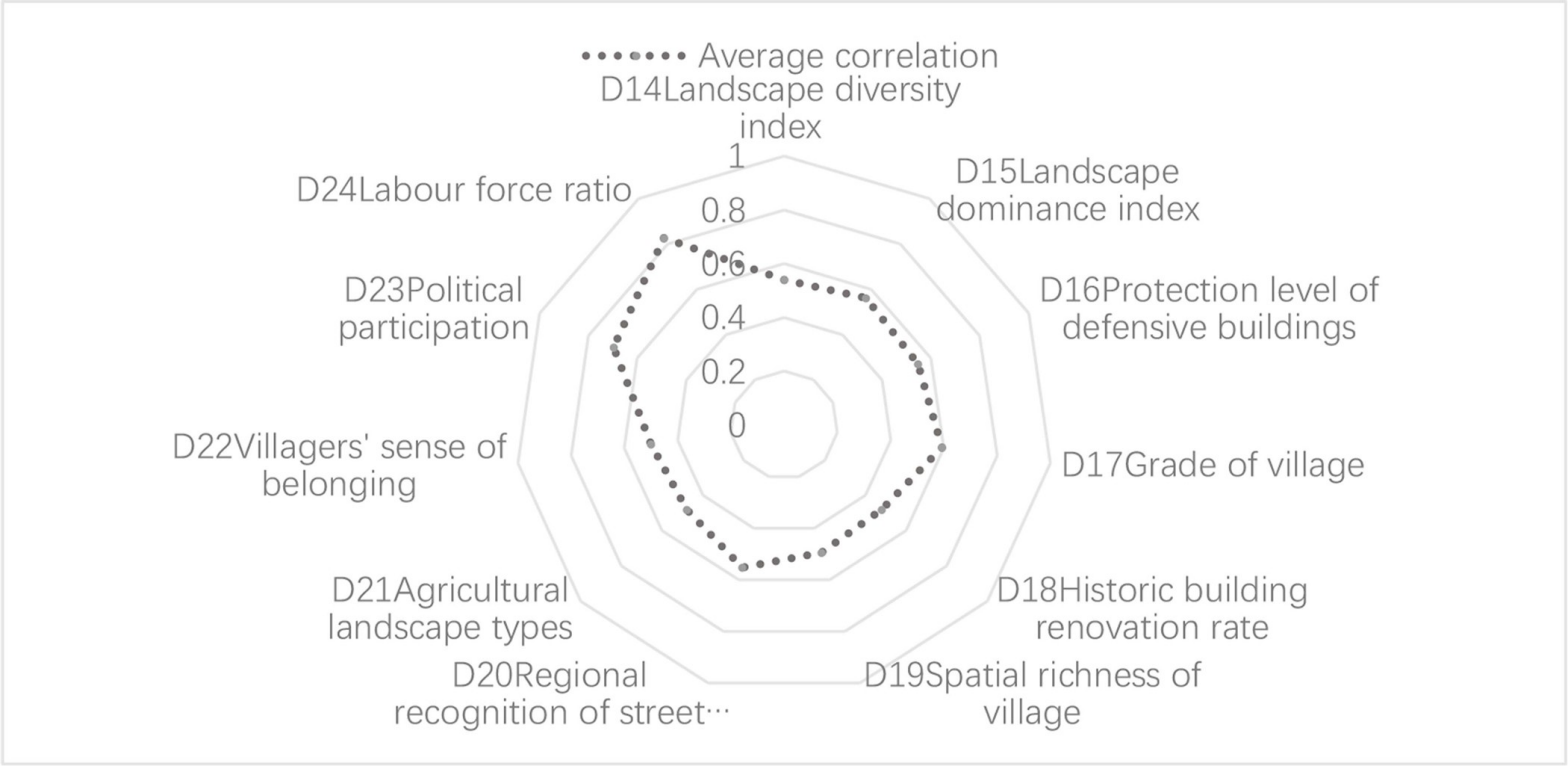
Correlation analysis of recovery of GWVs in Chicheng County.

In the recovery dimension, according to **Fig 12**, the high correlation between the indexes of political participation (D23) and labor force proportion (D24) indicates that two factors influence the CLRGWVs most. D24 (labor force ratio) refers to the proportion of the working population in the total population. The main workforce aged 15–64 years has greater learning capacity, which will promote the development of local industries and increase the capacity for recovery for villages. The high correlation of D23 political participation indicates that inhabitants are willing to participate in activities held by the local government, which is conducive to improving the social cohesion of the village. Secondly, village grade (D17), landscape dominance index (D15), and regional recognition of street landscape (D20) have a relatively positive impact on the degree of conservation in the village. In contrast, historic building renovation utilization (D18) and agricultural landscape type (D21) have a slight influence on resilience, suggesting that they have a relatively positive effect on the conservation of the GWVs. Overall, the GWVs have made some progress in terms of social cohesion; however, architecture conservation and ecological landscape diversity still need to be strengthened.

#### 4.3.3. Key influencing factors of learning

**Fig 13:**
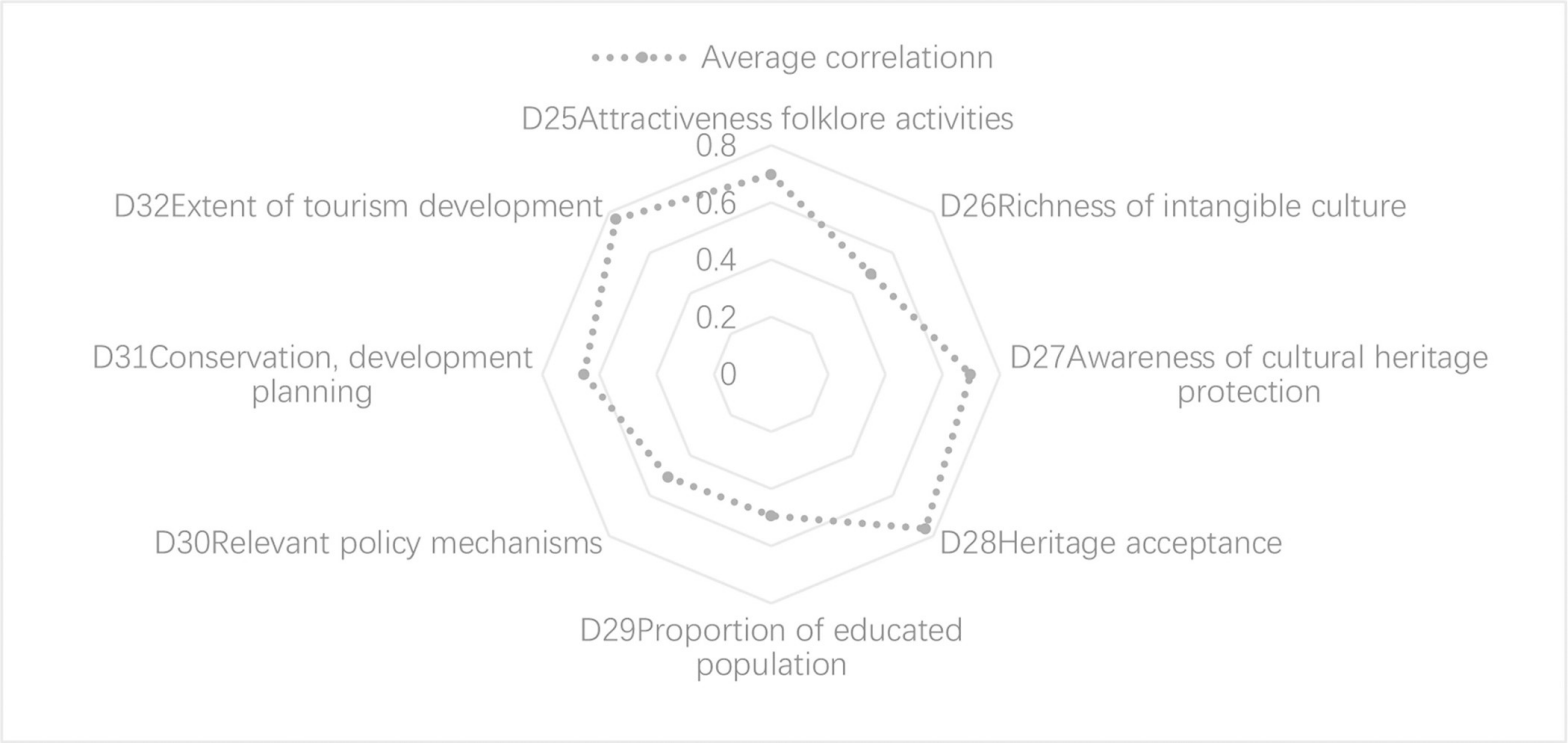
Correlation analysis of learning of GWVs in Chicheng County.

In the learning dimension, villager participation and the learning ability of communities, as reflected by the degree of heritage acceptance (D28) and the extent of tourism development (D32), have a significant positive impact on learning resilience. The extent of tourism development (D32) reflects the degree of systematic retrofit of the village, which reflects the management capacity of the local government and its ability to plan. Therefore, the village has a strong ability to deploy resources in the face of external disturbances. D28 Heritage acceptance indicates the level of awareness and conservation of heritage among villagers. The greater the awareness, the greater the participation of the villagers in the conservation of cultural landscapes, which is conducive to improving learning capacity. Moreover, the attractiveness of folklore activities (D25), awareness of cultural heritage conservation (D27), and conservation and development planning (D31) have also had a positive influence on learning resilience. Conversely, other indicators, such as the richness of intangible cultural heritage (D26) and the proportion of educated people (D29), have a lower correlation and weaker impact. The above results suggest that the collective participation of villagers and community learning contribute to the overall resilience level, but historical and cultural inheritance need to be reinforced in order to increase the level of learning resilience (Fig 13).

## 5. Discussion

### 5.1. Optimization principles and ideas of cultural landscapes of Great Wall Villages

#### 5.1.1. Principles

The first principle is wholeness. In terms of the Great Wall of China, it is more than a wall, it is a holistic military defense system, consisting of wall system, settlement system and information transmission system. Accordingly, the Great Wall needs a holistic and systematic conservation strategy. In terms of the construction of a resilience evaluation system, it is based on wholeness in systematics. The CLGWVs in the Chicheng region are viewed as an organic whole composed of social and ecological systems. The ecological and natural environment, historical, and cultural landscape resources need to be protected as a whole. The second principle is dynamicity. From perspective of Great Wall, it plays different roles at different stages in history. In Ming dynasty, it played the role of military defense. Nowadays, it is not only cultural landscape, but also symbols of military culture. It dynamically adapts to the changing times. Consequently, the Great Wall needs a dynamic conservation strategy to maintain the heritage itself and pass on the Great Wall culture. From a resilience perspective, the evolutionary process of cultural landscapes is non-linear, and it is necessary to focus on the inheritance of cultural ecosystems. Therefore, the dynamic and adaptive resilience optimization approach should be explored to cope with external threats, and multiple objectives should be dynamically coordinated. The third principle is continuity. The GWVs evolved from the military settlements of the Great Wall, which served as a military cantonment, and the defensive facilities have evolved into a unique defensive landscape. The CLGWVs have always evolved sustainably. As a result, this paper puts emphasis on the continuity of the original function of CLGWVs, the human-land relationship between the community and landscape, and the unity and continuity between the natural landscape and human landscape.

#### 5.1.2. Optimization ideas

The resilience optimization measures are based on the resilience evaluation index system of CLGWVs, the resilience evaluation results, and the results of key indicator identification. And the optimization strategies are proposed hierarchically according to the index levels. First, for the first level of indicators target, balancing resistance, recovery, and learning from a holistic perspective aims to improve the overall resilience level of the CLGWVs, which is the overall goal of resilience optimization. Second, for the second-level indicators criteria, the optimization objectives of maintaining resistance, enhancing recovery, and elevating learning ability are proposed based on the product of the indicator weights of resistance, recovery, and balance and the evaluation scores of cases. Third, for factor layers, in terms of resistance, recovery, and learning, the indicators with lower evaluation results are prioritized for improvement, and optimization measures are given accordingly. For example, in terms of resistance, all three villages perform poorly in terms of stability of ecological environment (C1) and stability of social structure (C4), so we should first adjust the ecological spatial pattern and improve the stability of social structure. Three GWVs perform better in both integrity of defensive villages (C2) and preservation of spatial patterns (C3), therefore, we should consolidate by building spatial networks. Finally, in terms of the index layer, based on the final evaluation results of each indicator and the identification results of key indicators, the priority order of indicator enhancement is jointly determined. Combined with the references, specific measures suitable for this study to ultimately enhance the relevant resilience indicators are proposed.

### 5.2. Maintain system’s resistance

#### 5.2.1. Construct a spatial network of cultural landscap

**Fig 14:**
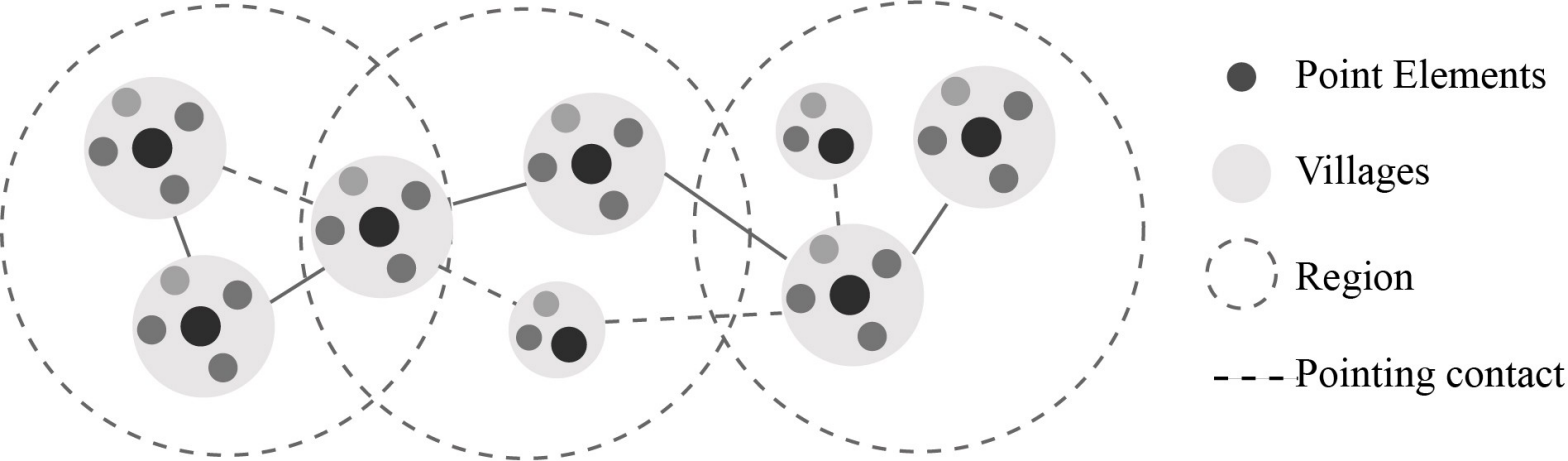
Schematic diagram of the spatial network of GWVs defense landscape. Small black dot represents point elements, big grey circle represents villages, dotted line represents region, dotted line represents pointing contact.

According to key influencing factors of resistance, the proportion of indigenous people in the total population (D12), the destruction degree of the remains (D4), and road accessibility (D10) have a greater impact on resistance levels. Based on the evaluation result of resistance, the low level of deterioration degree of the remains (D4) and road accessibility (D10) reflect that connectivity of the cultural landscape within the village is poor and a number of interconnected landscape nodes need to be integrated. Additionally, the defensive landscape of GWVs is characterized by a certain linear cultural heritage and is composed of several landscape nodes, which can form a specific system of spatially integrated networks. As is discussed by Bin Feng, it is a spatial network shaped by the combination of closely linked corridors and patches for conservation and utilization [70]. Therefore, a spatial integration network to resist external disturbances should be built. Firstly, various types of landscape nodes should be linked in series to improve accessibility. Grade and classify cultural landscape elements. Secondly, utilize the natural environment to form the base environment. Then, use transportation to integrate the nodes of the landscape. Additionally, establishing a visual link between the village and the defensive landscape is conducive to improving connectivity (Fig 14).

#### 5.2.2. Adjust the ecological spatial pattern

In terms of the low level of landscape patch density (D2), ecological structure needs to be adjusted to enhance the stability of the ecological environment. Therefore, it is essential to construct ecological spatial networks. As discussed by Shi Qiu, an ecological spatial network consists of ecological nodes and ecological corridors [71]. Longmensuo village has a high landscape dominance index and vegetation cover but relatively low stability in its ecological pattern. So build an ecological park along the Bai River, combining it with defensive landscapes. Construct landscape nodes through greenery and plazas to enrich the types of landscapes.

#### 5.2.3. Strengthen the stability of the social structure

The low level of the proportion of indigenous people (D12) indicates that social structure is unstable. Additionally, D12 has the greatest influence on resistance level, so it is critical to optimize demographic structure. For Ningyuanbao village with heavy population loss, from the government’s perspective, it is necessary to implement talent introduction strategies and improve the talent service system to attract and retain talent. In addition, from the perspective of the villagers, it is critical to increase their participation in the development and construction of the village. To carry out a wide range of activities, such as forming a volunteer community Great Wall guard to protect the defensive landscape and organizing celebrations to improve adaptive capacity and learning ability. Similar strategies, like building community networks, have been discussed by Mohammad Mojibul Hoque Mozumder [72].

### 5.3. Enhance recovery capability of system

#### 5.3.1. Diversify elemental functions

According to the evaluation results of Recovery, all three villages perform worst in the diversity of landscape patterns. Different from two other villages, Ningyuanbao village only has residential buildings. A single function leads to lower resilience. So to make landscape patterns diverse, firstly, attention should be paid to maintaining the original function of historical buildings. Clarify the protection level of traditional buildings and renovate those with high historical value. Secondly, new functions should be appropriately implanted to activate villages and meet the diverse needs of villagers.

#### 5.3.2. Continue street fabric

The low level of spatial richness of the village (D19) and regional recognition of the street landscape (D20) reflect the importance of continuing the morphological fabric of the village. Furthermore, street structure is an important component of landscape pattern. Because of the great variation in village form and the low degree of morphological integrity preserved, the street texture of Ningyuanbao village is subsequently highly variable. So it is essential to connect the Great Wall, historical buildings, and other iconic landscapes with the street to increase visual connection. Moreover, control the street scale, facade form, and paving of the roads.

#### 5.3.3. Pass on social and cultural heritage

According to the evaluation result of recovery, the low degree of villagers’ sense of belonging (D22) influences recovery capacity. Villagers are important participants in the development of social culture and the main carriers of social culture. As shown in the study, encouraging community participation in local social organization activities is a critical social resilience element [73]. Therefore, it is crucial to increase villagers’ recognition of traditional culture, participation in folklore activities, and value identification. The government should provide reasonable guidance to create a humanistic environment in the GWVs.

### 5.4. Elevate the learning ability

#### 5.4.1. Establish resilient management mechanism

In response to the low level of relevant policy mechanisms (D30) in the dimension of human landscape management, it is necessary to establish effective and resilient management mechanisms on the basis of the relationship between cultural landscape elements and their historical and cultural values. In the dimension of social development, make full use of the cultural landscape resources to carry out rational planning. A similar strategy has been discussed by Dastgerdi, who explored the way urban governance and policy provide room for enhancing cultural landscape resilience against natural hazards. They discovered cultural landscapes require a bottom-up, participatory structure [48].

#### 5.4.2. Enhance social self-organization

Community organizational capacity is the mainstay of the village system’s learning ability and the basis for enhancing social resilience. Therefore, the coordination of top-down and bottom-up models can strengthen public participation and enhance social cohesion. Secondly, from the perspective of experts and the government, mainly through management and coordination, work with villagers to strengthen the construction of resilient communities.

### 5.5. Contributions and implications

The issue that the cultural landscape in GWVs was focused on, generally with an undeveloped economy and mountainous terrain, is prone to falling into the double dilemma of natural disasters and social shock. The contributions and implications of this study are as follows: First, apply resilience theory to the evaluation of cultural landscapes and construct a resilience evaluation system. Then, through the resilience evaluation of Ningyuanbao Village, Longmensuo Village, and Dushikou Village, we find their advantages and disadvantages in resilience. For example, in Ningyuanbao Village, the spatial pattern is well preserved, and the village is less shocked by modern development but has single productive and living landscapes. Dushikou Village has a higher level of social resilience, especially in community learning, but its ecological space is severely taken up by modern construction. Longmensuo Village’s defensive landscape and spatial pattern are well preserved, but socio-cultural and demographic structures need improvement. For all GWVs, the resilience of recovery needs to be promoted, especially in terms of diversity of landscape patterns and diversity of production space. The findings of the evaluation help validate the rationality of the resilience evaluation system. Second, compared with previous studies that only considered single and static protection strategies, this study constructed a resilience evaluation system, focusing on the resilience stage characteristics of the cultural landscape within the villages. So the conservation of cultural landscapes is a dynamic process, providing a framework for resilience management and new ideas for cultural landscape conservation research. Third, the theory of resilience provides new ideas and directions for dealing with the sustainable development of villages and the conservation of cultural landscapes. The study of the CLREGWVs will help villages deal with the relationship between socio-economic development and the conservation of cultural landscapes.

#### 5.6. Limitations

Although this study constructed the resilience evaluation index system and analyzed the resilience of three GWVs, there are still some limitations: (1) The basic limitations of the study stem from the method used. In the process of using AHP, whether building a hierarchy or constructing a judgment matrix, subjective judgment, choices, and preferences may have an impact on the results. With regard to evaluation, the experience and knowledge of experts may have an effect on evaluation results. When it comes to GRA, it is not suitable for wide application as it is more subjective when judging the optimal value. Therefore, the methods are subjective to a certain extent, which may affect the scientificity of the research results. (2) The second limitation results from the selection of the sample. There are 22 GWVs in Chicheng County, but restricted by time, experience, and the complexity of the GWVs, only three villages covering different scales (military grade and economic level) were selected to conduct case studies. It is not conducive to summarize the resilient characteristics and put forward more scientific optimization strategies. In subsequent studies, it is essential to appropriately supplement the number of villages and broaden the scope of cases. (3) The third limitation results from the selection of indicators. There are many influencing factors involved in the construction of a resilience evaluation system. In addition, due to the lack of a unified resilience factor assignment standard, the research refers to relevant studies and combines them. There are still some factors that are not considered.

## 6. Conclusions

In the context of the rural revitalization strategy, the GWVs confront with more and more opportunities and challenges. On the one hand, the CLGWVs is vulnerable to the external disturbances. The destruction of the landscape, the disappearance of traditional culture and the ageing of the social structure threaten the villages with decay. On the other hand, with their advantage in geographic location and natural environment, GWVs have the potential to build resilience system.

Therefore, this study constructsa cultural landscape resilience evaluation index system for the GWVs and conducts evaluation in order to realize the sustainable development of GWVs.The main findings of this paper are as follows:

### (1) The resilience evaluation system of CLGWVs was constructed

Firstly, this paper innovatively tries to apply resilience theory to the analysis of the CLGWVs on the basis of analysis of suitability and feasibility of this theory. Secondly, an interconnected framework between resilience theory and CLGWVs was constructed through the two dimensions of "element association" and "feature association". Lastly, a resilience evaluation system for CLGWVs was established with the method of AHP.

### (2) The resilience evaluation system of cultural landscape is reasonable and applicable to the GWVs in China

Case study of three GWVs verified the rationality of the evaluation system. Taking Chicheng County as the study area, three representative Great Wall Villages, Ningyuanbao, Dushikou and Longmensou, are selected to conduct resilience investigation and analysis, and evaluation results are obtained and dissected to further prove the rationality and applicability of the evaluation system.

### (3) The resilience evaluation system of cultural landscape contributes to the sustainable development of the GWVs in China

Under the principles of wholeness, dynamicity and continuity, based on the dual results of the resilience evaluation results and key indicator analysis, the objectives of resilience optimization are determined hierarchically, and appropriate resilience optimization countermeasures are proposed by prioritizing the improvement of indicators and referring to the existing researches.

## Supporting information

S1 Fig Cultural landscape in Ningyuanbao Village. (A) Geographical location of Ningyuanbao Village. The base map was from USGS EROS: http://eros.usgs.gov/#. Yellow line reprsents fort, blue line represents river, orange line represents the Great Wall. (B) Current spatial layout of Ningyuanbao Village. The figure was made by Weiya Zhang. The thinnest light gray line represents streets, pink area represents original site area, blue-gray line represents external transport, dark gray line represents national roads, the light blue areas represent rivers. (C) The Great Wall called Dichangcuo. (D) Eastern beacon tower. (E) Beacon tower called Shuiguan. (F) Southern gate of Ningyuanbao Fortress. (G) Beacon tower at Ningyuanbao Village. The photos of (C)-(G) were provided by Xiaodong Ming.
(PDF)

S2 Fig Cultural landscape in Dushikou Village. (A) Geographical location of Dushikou Village. The base map was from USGS EROS: http://eros.usgs.gov/#. Yellow line reprsents fort, blue line represents river, orange line represents the Great Wall. (B) Current spatial layout of Dushikou Village. The figure was made by Weiya Zhang. The thinnest light gray line represents streets, pink area represents original site area, blue-gray line represents external transport, dark gray line represents national roads, the light blue areas represent rivers. (C) The Great Wall in Dushikou Village. (D) The southern wall of Dushikou Fortress. (E) Beacon tower. (F) Pailou at western gate of Dushikou Village. (G) Rock called Dushi. The photos of (C)-(G) were provided by Xiaodong Ming.
(PDF)

S3 Fig Cultural landscape in Longmensuo Village. (A) Geographical location of Longmensuo Village. The base map was from USGS EROS: http://eros.usgs.gov/#. Yellow line reprsents fort, blue line represents river, orange line represents the Great Wall. (B) Spatial layout of Longmensuo Village. The figure was made by Weiya Zhang. The thinnest light gray line represents streets, pink area represents original site area, blue-gray line represents external transport, dark gray line represents national roads, the light blue areas represent rivers. (C) Wall remains of Longmensuo Fortress. (D) Outer citadel of Longmensuo Fortress. The photos of (C) and (D) were provided by Xiaodong Ming.
(PDF)

S1 Table Basic information of three GWVs. (PDF)

S2 Table Scoring table for the weights of indicators for evaluating the resilience of the CLGWVs. (PDF)

S3 Table Indices of the cultural landscape resilience of GWVs. (PDF)

S4 Table Questionnaire of cultural landscape resilience evaluation of Great Wall Villages. (PDF)

S5 Table The resilience evaluation result of CLGWVs. (PDF)
