# Supplementary figures and images for: Cultural landscape resilience evaluation of Great Wall Villages: A case study of three villages in Chicheng County
Source: PLoS One. 2024 Apr 18;19(4):e0298953. doi: 10.1371/journal.pone.0298953 (PMC11025826; doi:10.1371/journal.pone.0298953)

A

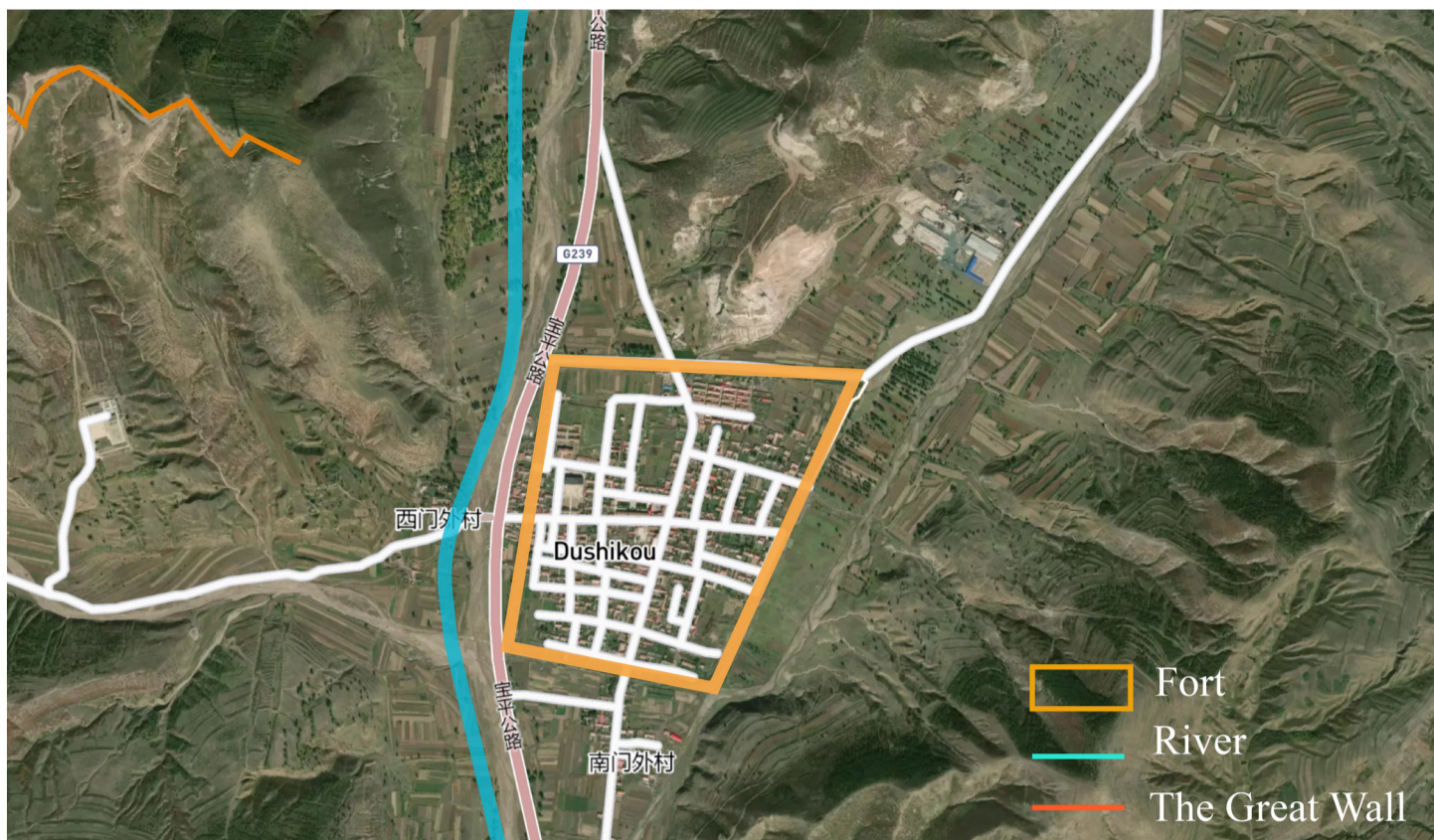

C

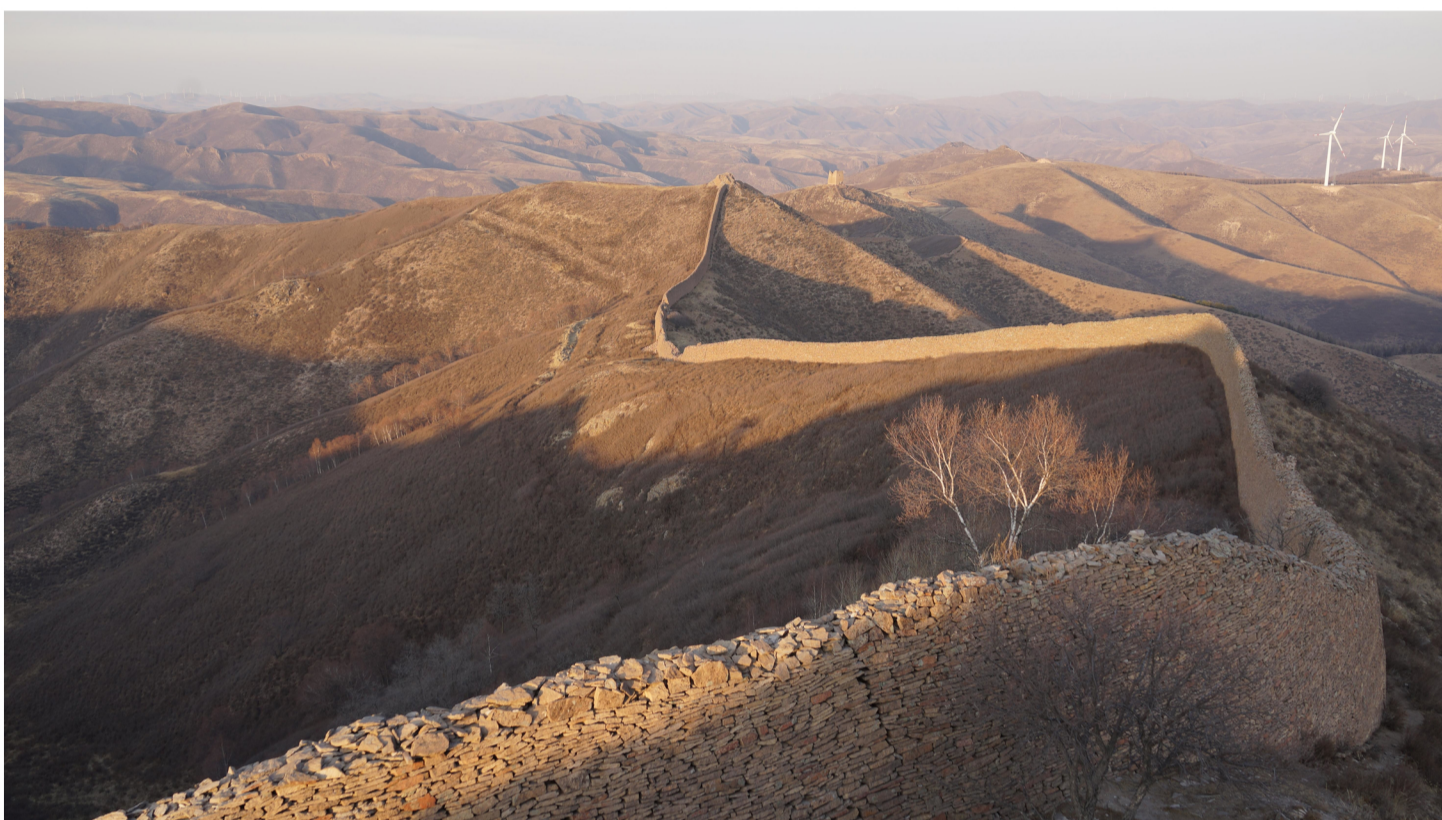

E

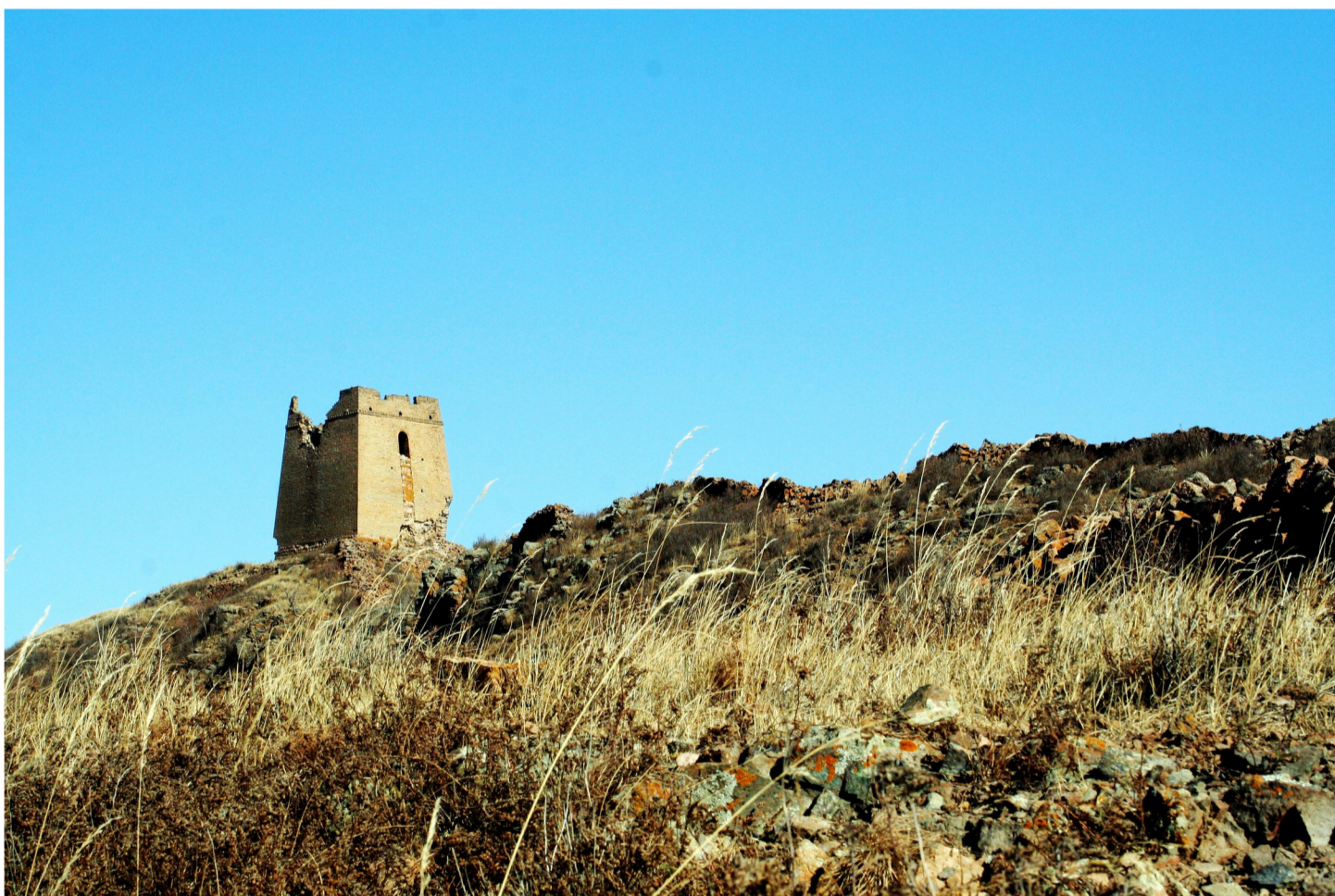

G

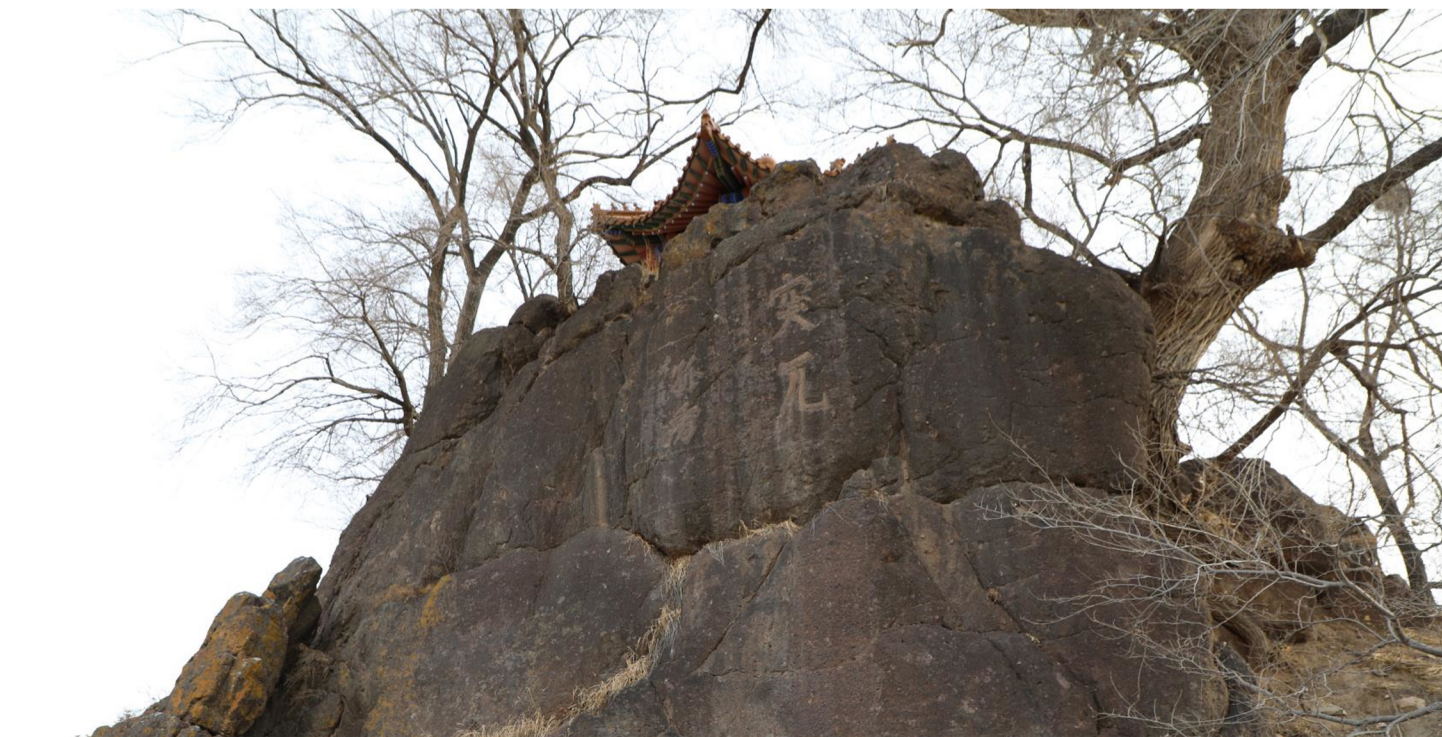

B

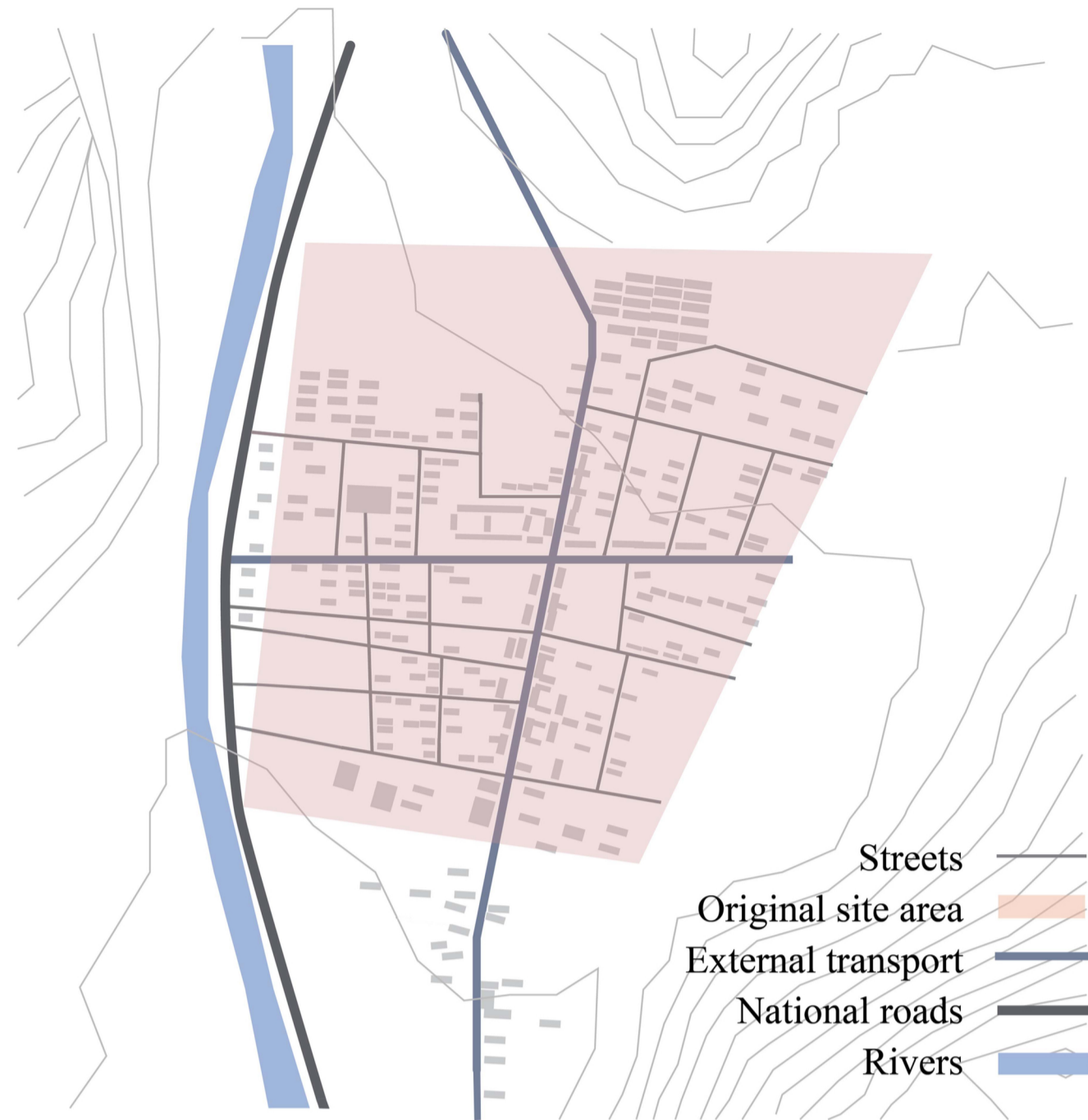

D

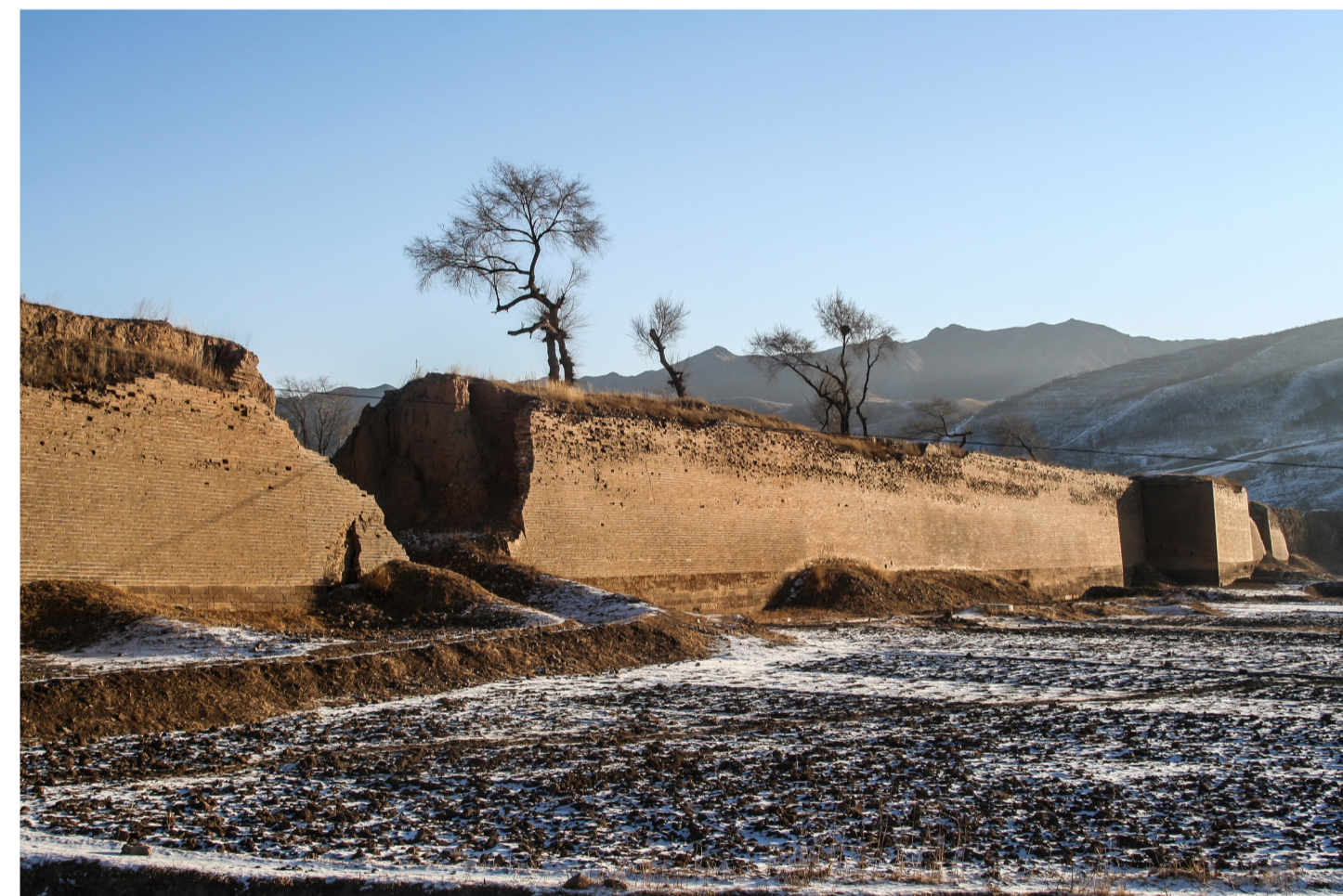

F

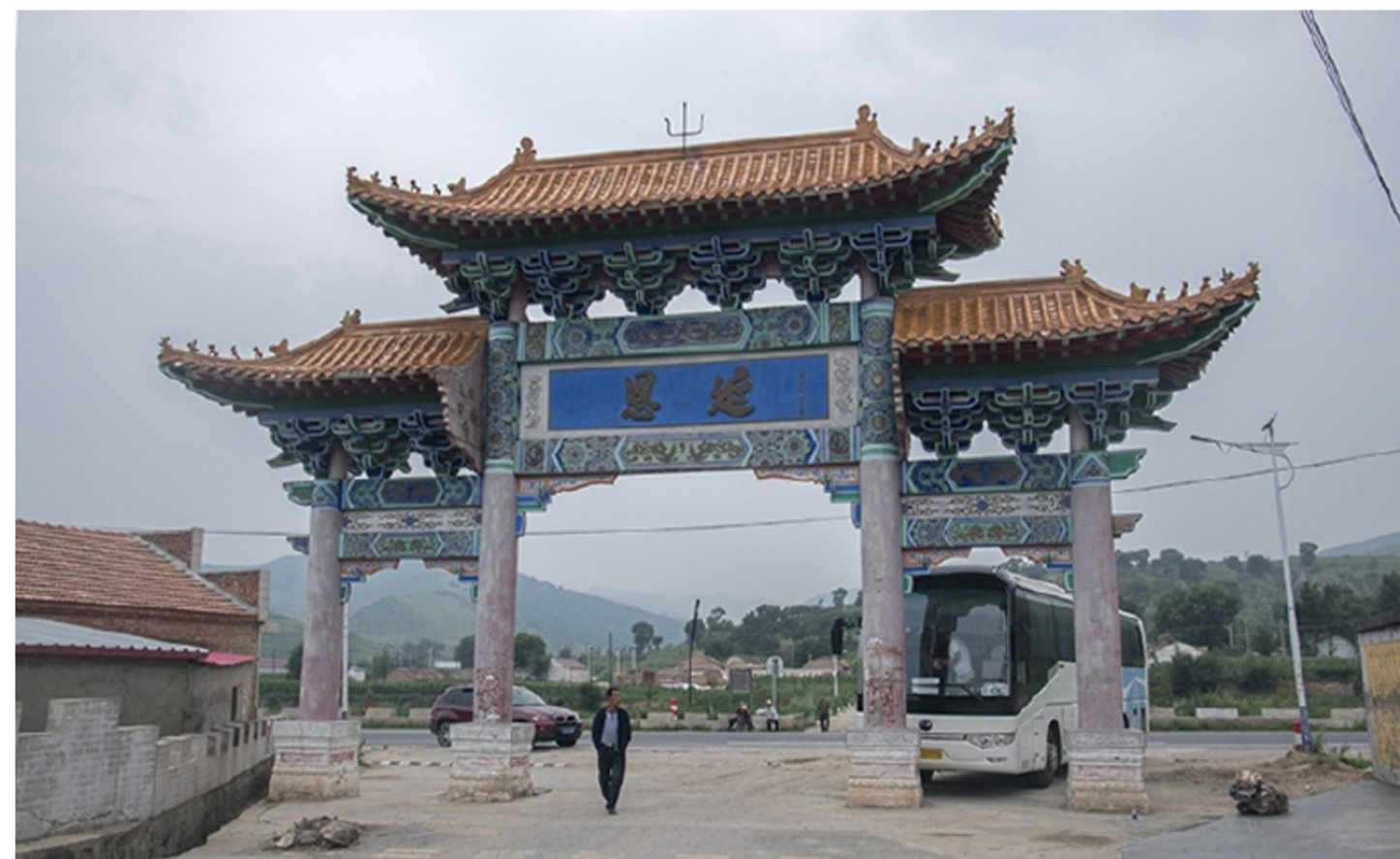

Supplement: S1 Fig — (A) Geographical location of Ningyuanbao Village. The base map was from USGS EROS: http://eros.usgs.gov/#. Yellow line reprsents fort, blue line represents river, orange line represents the Great Wall. (B) Current spatial layout of Ningyuanbao Village. The figure was made by Weiya Zhang. The thinnest light gray line represents streets, pink area represents original site area, blue-gray line represents external transport, dark gray line represents national roads, the light blue areas represent rivers. (C) The Great Wall called Dichangcuo. (D) Eastern beacon tower. (E) Beacon tower called Shuiguan. (F) Southern gate of Ningyuanbao Fortress. (G) Beacon tower at Ningyuanbao Village. The photos of (C)-(G) were provided by Xiaodong Ming. (PDF) [file pone.0298953.s001.pdf]

A

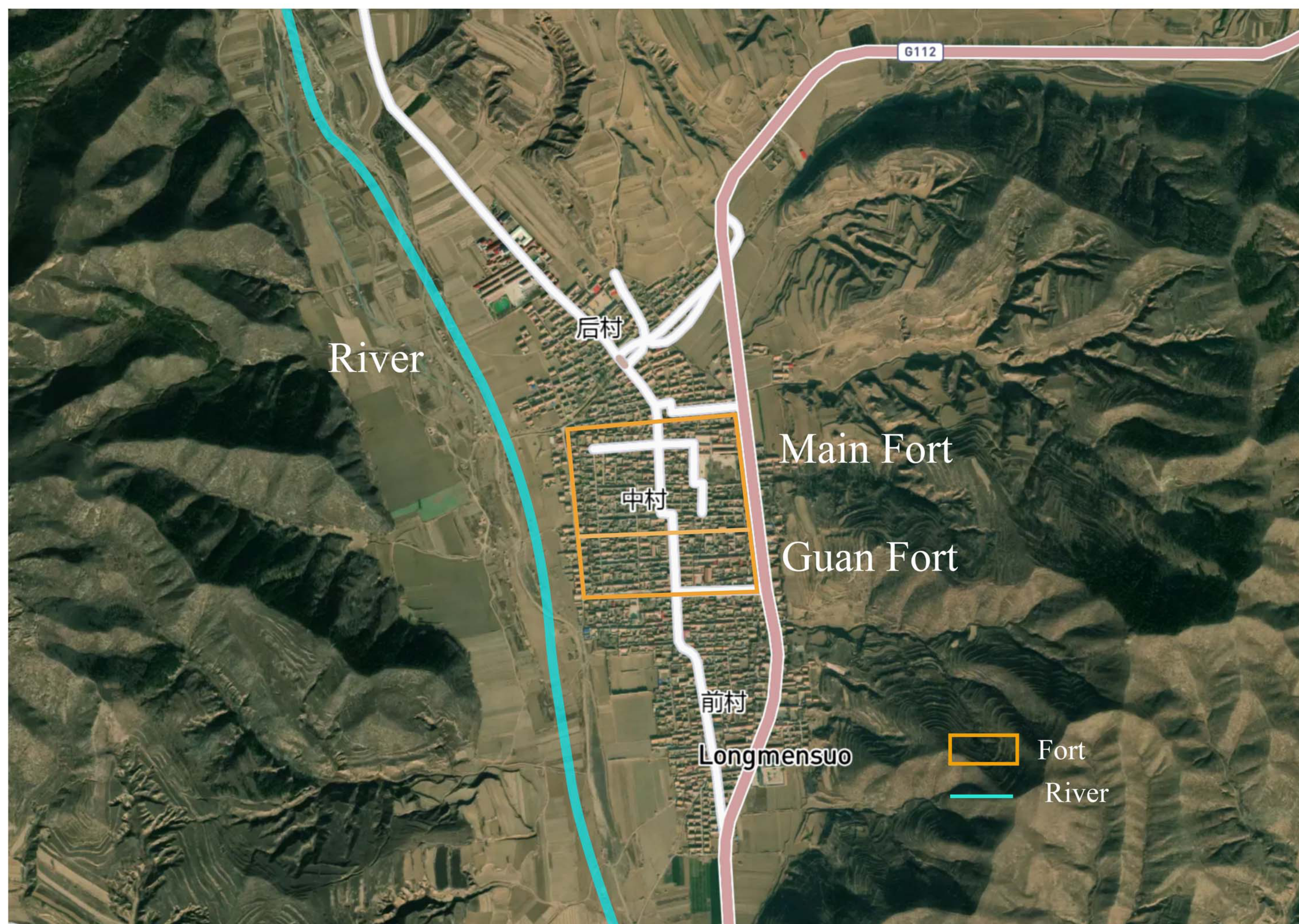

B

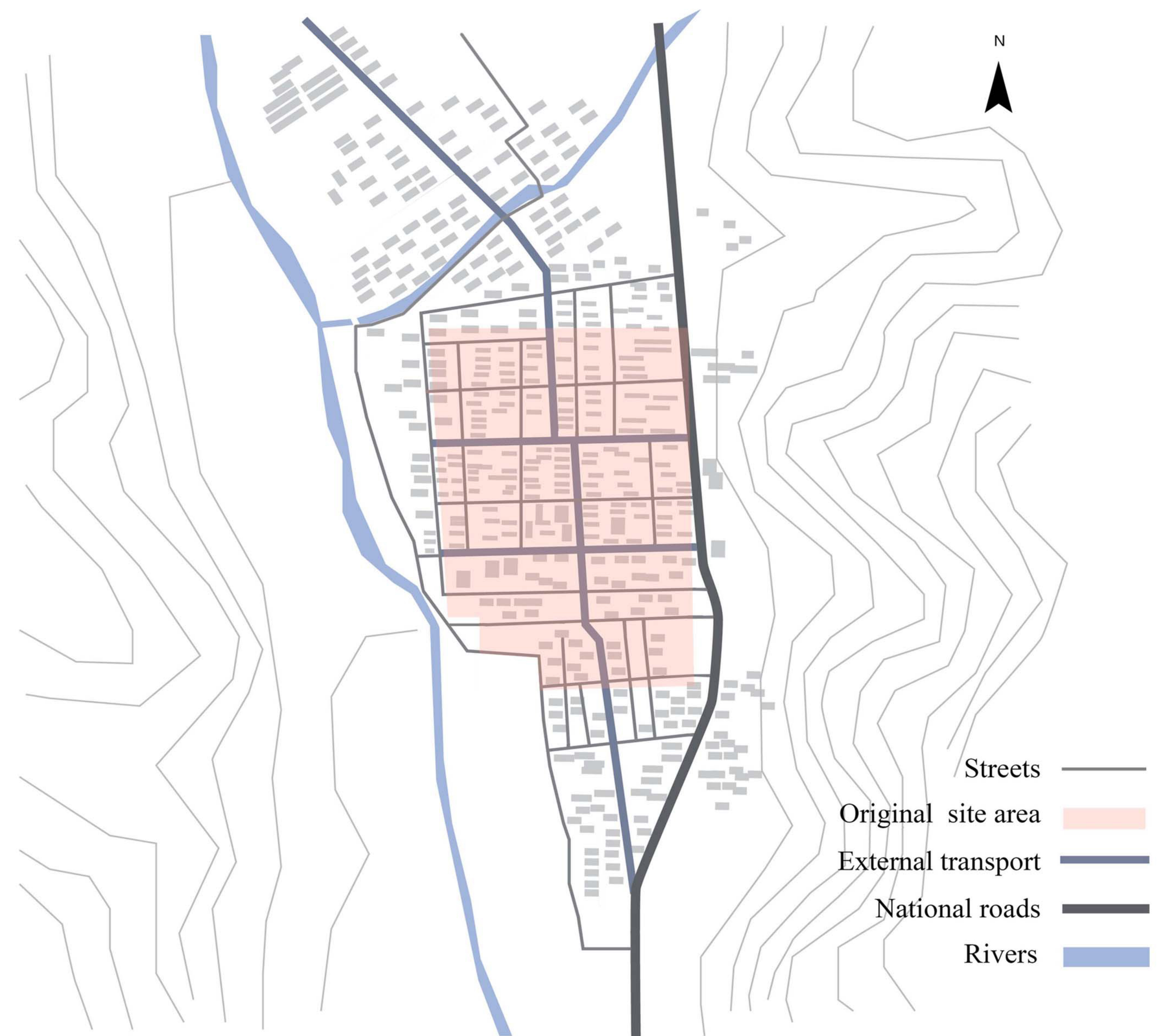

C

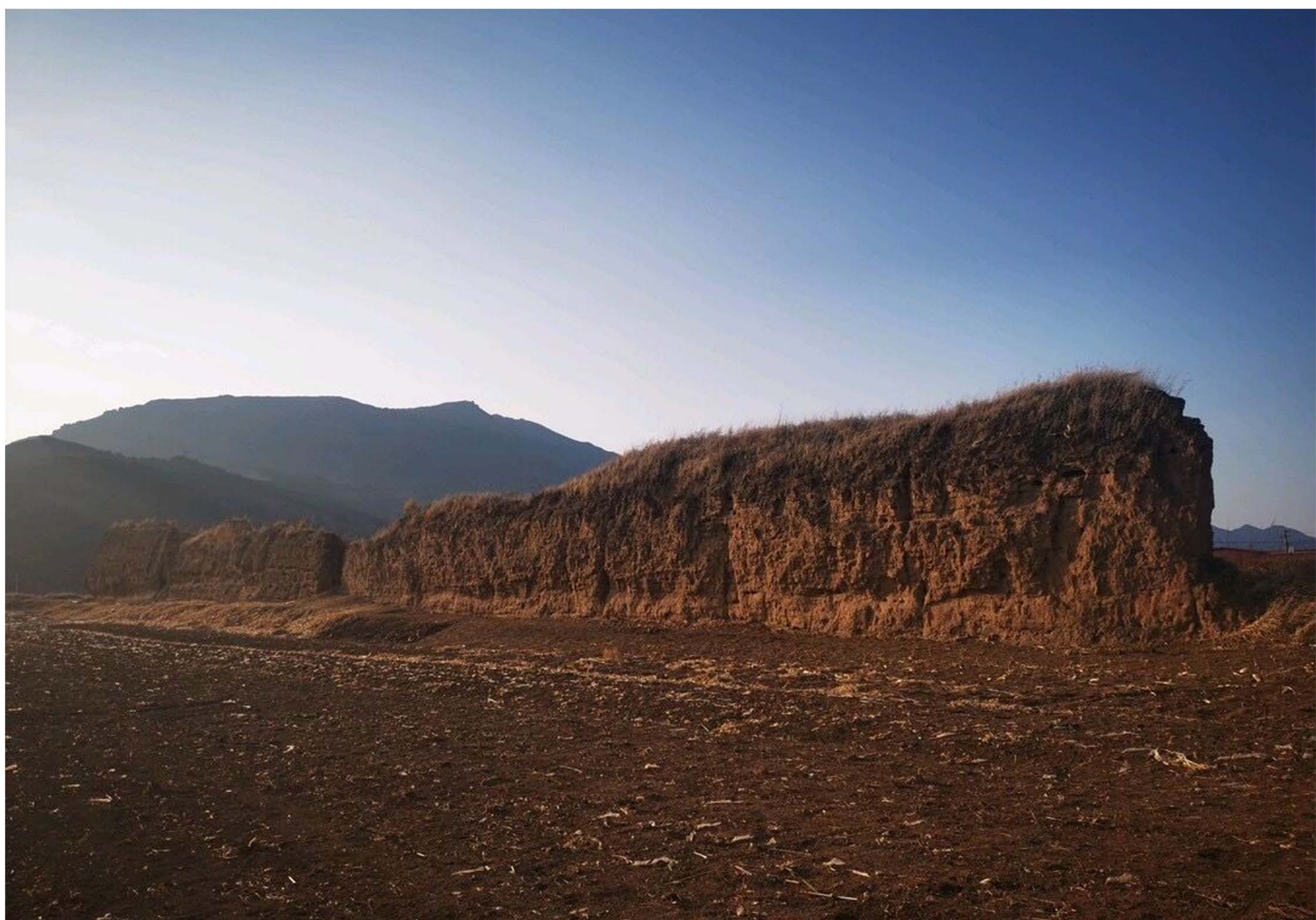

D

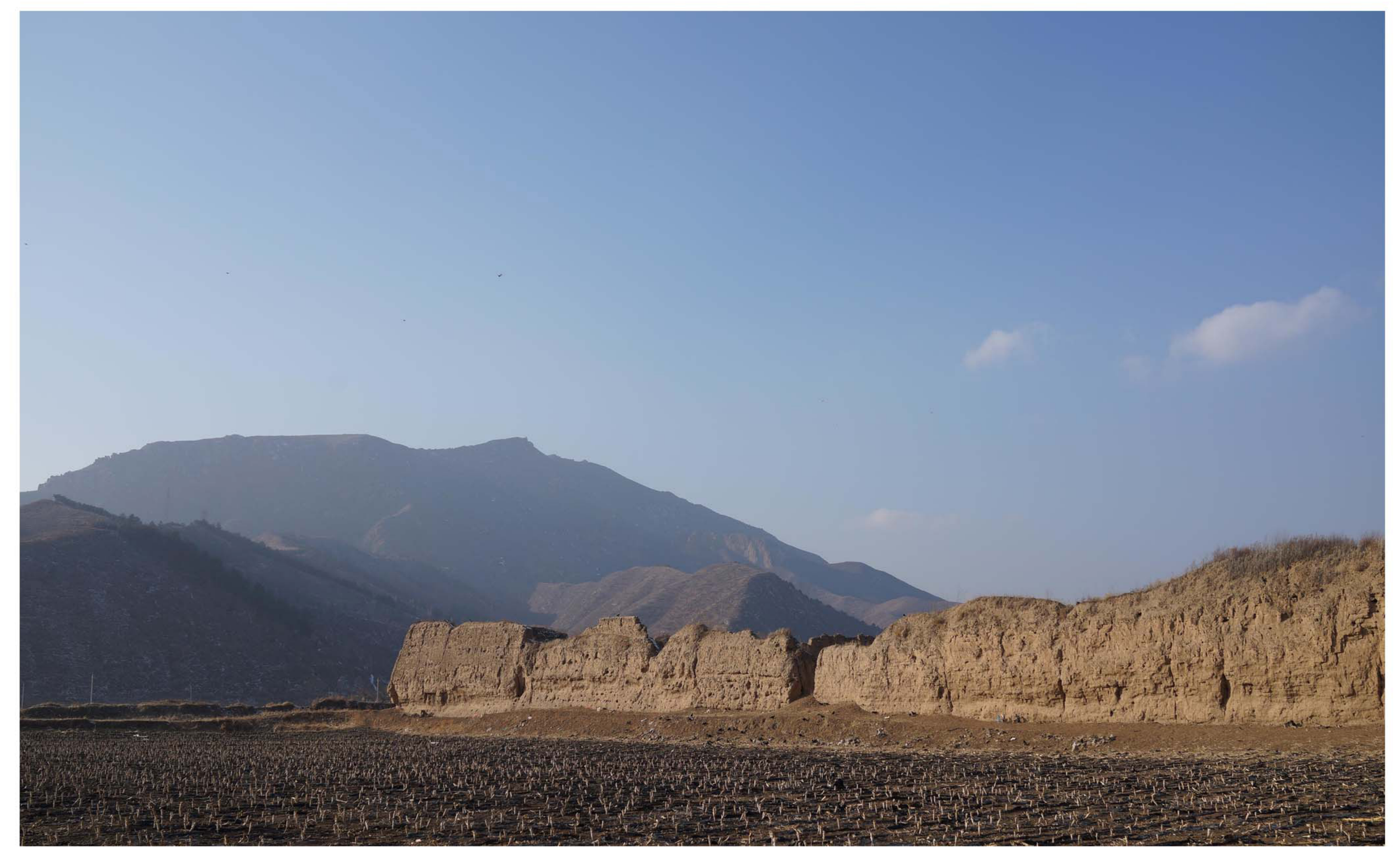

Supplement: S3 Fig — (A) Geographical location of Longmensuo Village. The base map was from USGS EROS: http://eros.usgs.gov/#. Yellow line reprsents fort, blue line represents river, orange line represents the Great Wall. (B) Spatial layout of Longmensuo Village. The figure was made by Weiya Zhang. The thinnest light gray line represents streets, pink area represents original site area, blue-gray line represents external transport, dark gray line represents national roads, the light blue areas represent rivers. (C) Wall remains of Longmensuo Fortress. (D) Outer citadel of Longmensuo Fortress. The photos of (C) and (D) were provided by Xiaodong Ming. (PDF) [file pone.0298953.s003.pdf]
