# Supplementary material for: Cultural landscape resilience evaluation of Great Wall Villages: A case study of three villages in Chicheng County
Source: PLoS One. 2024 Apr 18;19(4):e0298953. doi: 10.1371/journal.pone.0298953 (PMC11025826; doi:10.1371/journal.pone.0298953)
Supplement: S1 Table — (PDF) [file pone.0298953.s004.pdf]

S1 Table. Basic information of Three Great Wall Villages

**Village name**	**Military grade**	**Natural Environment**			**Defensive buildings**				**Spatial pattern**	**Public facilities**
		Village area/ km²	Forest coverage	patch density	Border wall/m	Beacon tower	Turrets	Watch tower		
Ningyuanbao	Bao Citadel	13.7	54.3%	324.64	1482	2	2	4	rectangular	Southern gate
Dushikou	Wei Citadel	11.3	37%	401.23	6547	0	2	0	Square	Dushi Temple, Yuhuang Ge, Zhangmao Mountain, Yiwanquan
Longmensuo	Suo Citadel	22.03	62%	61.33	0	0	0	2	Ribbon	Guandi Temple Mashen Temple Wen Temple
