# Supplementary material for: Cultural landscape resilience evaluation of Great Wall Villages: A case study of three villages in Chicheng County
Source: PLoS One. 2024 Apr 18;19(4):e0298953. doi: 10.1371/journal.pone.0298953 (PMC11025826; doi:10.1371/journal.pone.0298953)
Supplement: S2 Table — (PDF) [file pone.0298953.s005.pdf]

Scoring table for the weights of indicators for evaluating the resilience of the cultural landscape of the Great Wall Villages

In order to determine the weight of each indicator in the evaluation system, this study adopts the Expert Scoring method and the Analytic Hierarchy Process (AHP) method to determine the weight of each indicator.

Firstly, the evaluation system of cultural landscape resilience of the Great Wall villages is as follows:

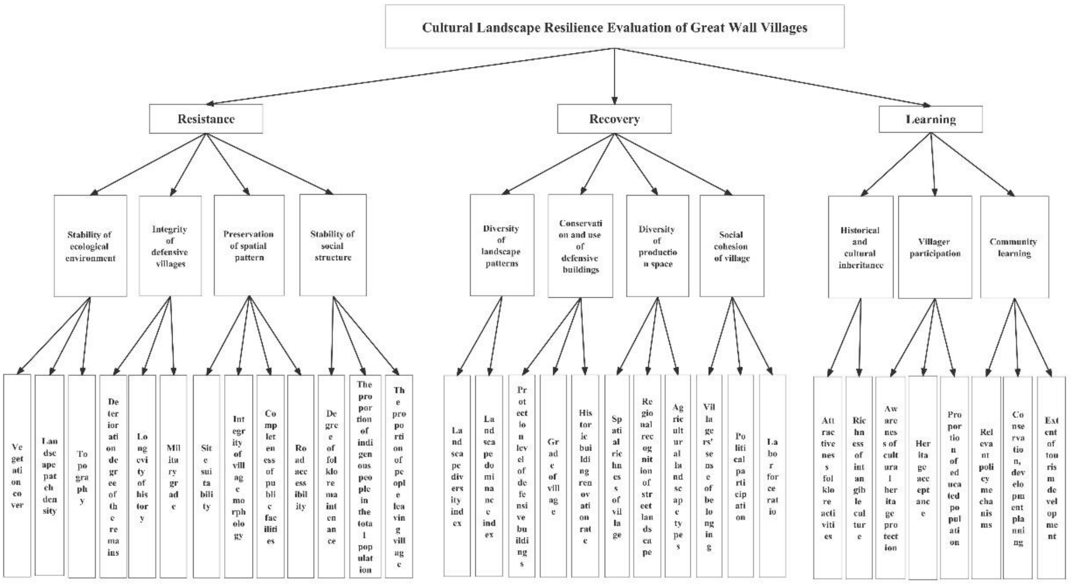

Secondly, during the process of determination of indicator weights, for indicators in the same layer, scores are assigned on an evaluation scale of 1-9, with each score defined as follows:(Table1)

Table1 Factor scale importance 1-9 scale.

Scale	1	3	5	7	9	2,4,6,8
Implication	Factors i,j are equally important	Factor i is slightly moreimportant than factor j	Factor i is significantly more important than factor j	Factor i is strongly more important	Factor i is extremely more	Intermediate values

		than factor j	importa nt than factor j
Reciproc al	αij is the comparison result of the importance of factor I and factor j, $\alpha ij=1/\alpha ij$		

Thirdly, Weighting Score Table (please fill in after the score, do not fill in if there is a / symbol in the table)

（1）Judgement matrix of indicators of criteria

Judgement matrix of indicators of criteria

Cultural Landscape Resilience Evaluation of GWVsA	ResistanceB1	RecoveryB2	LearningB3
ResistanceB1	1	Score：	Score：
RecoveryB2	/	1	Score：
LearningB3	/	/	1

（2）Judgement matrix of indicators of Factor Layer

Judgement matrix of B1 Resistance

ResistanceB1	Stability of ecological environmentC1	Integrity of defensive villagesC2	Preservation of spatial patternC3	Stability of social structureC4
Stability of ecological environmentC1	1	Score：	Score：	Score：
Integrity of defensive villagesC2	/	1	Score：	Score：
Preservation of spatial patternC3	/	/	1	Score：
Stability of social structureC4	/	/	/	1

Judgement matrix of B2 Recovery

RecoveryB2	Diversity of landscape patterns C5	Conservation and use of defensive buildingsC6	Diversity of production spaceC7	Social cohesion of villageC8
Diversity of landscape patterns C5	1	Score：	Score：	Score：
Conservation and use of defensive buildingsC6	/	1	Score：	Score：
Diversity of production spaceC7	/	/	1	Score：
Social cohesion of villageC8	/	/	/	1

Judgement matrix of B3 Learning

LearningB3	Historical and cultural inheritance C9	Villager participation C10	Community learning C11
Historical and cultural inheritance C9	1	Score：	Score：
Villager participationC10	/	1	Score：
Community learningC11	/	/	1

（3）Judgement matrix of indicators of Index Layer

Judgement matrix of C1 Stability of ecological environment

Stability of ecological environmentC1	Vegetation coverD1	Landscape patch densityD2	Topography D3
Vegetation coverD1	1	Score：	Score：
Landscape patch densityD2	/	1	Score：
Topography D3	/	/	1

Judgement matrix of C2 Integrity of defensive villages

Integrity of defensive villagesC2	Deterioration degree of the remainsD4	Longevity of historyD5	Military gradeD6

Deterioration degree of the remainsD4	1	Score:	Score:
Longevity of historyD5	/	1	Score:
Military gradeD6	/	/	1

Judgement matrix of C3 Preservation of spatial pattern

Preservation of spatial patternC3	Site suitability D7	Integrity of village morphology D8	Completeness of public facilitiesD9	Road accessibility D10
Site suitability D7	1	Score:	Score:	Score:
Integrity of village morphology D8	/	1	Score:	Score:
Completeness of public facilitiesD9	/	/	1	Score:
Road accessibility D10	/	/	/	1

Judgement matrix of C4 Stability of social structure

Stability of social structureC4	Degree of folklore maintenanceD11	The proportion of indigenous people in the total population D12	The proportion of people leaving village D13
Degree of folklore maintenanceD11	1	Score:	Score:
The proportion of indigenous people in the total population D12	/	1	Score:
The proportion of people leaving village D13	/	/	1

Judgement matrix of C5 Diversity of landscape patterns

Diversity of landscape patterns C5	Landscape diversity index D14	Landscape dominance index D15
Landscape diversity index D14	1	Score:
Landscape dominance index D15	/	1

Judgement matrix of C6Conservation and use of defensive buildings

Conservation and use of defensive buildingsC6	Protection level of defensive buildingsD16	Grade of villageD17	Historic building renovation rate D18
Protection level of defensive buildingsD16	1	Score：	Score：
Grade of villageD17	/	1	Score：
Historic building renovation rate D18	/	/	1

Judgement matrix of C7 Diversity of production space

Diversity of production spaceC7	Spatial richness of village D19	Regional recognition of street landscape D20	Agricultural landscape types D21
Spatial richness of village D19	1	Score：	Score：
Regional recognition of street landscape D20	/	1	Score：
Agricultural landscape types D21	/	/	1

Judgement matrix of C8 Social cohesion of village

Social cohesion of village C8	Villagers' sense of belonging D22	Political participation D23	Labor force ratio D24
Villagers' sense of belonging D22	1	Score：	Score：
Political participation D23	/	1	Score：
Labor force ratio D24	/	/	1

Judgement matrix of C9 Historical and cultural inheritance

Historical and cultural inheritance C9	Attractiveness folklore activities D25	Richness of intangible culture D26
Attractiveness folklore activities D25	1	Score：
Richness of intangible culture D26	/	1

Judgement matrix of C10 Villager participation

Villager participation C10	Awareness of cultural heritage protection D27	Heritage acceptance D28	Proportion of educated population D29
Awareness of cultural heritage protection D27	1	Score：	Score：
Heritage acceptance D28	/	1	Score：
Proportion of educated population D29	/	/	1

Judgement matrix of C11 Community learning

Community learning C11	Relevant policy mechanisms D30	Conservation, development planning D31	Extent of tourism development D32
Relevant policy mechanisms D30	1	Score：	Score：
Conservation, development planning D31	/	1	Score：
Extent of tourism development D32	/	/	1
