# Supplementary material for: Cultural landscape resilience evaluation of Great Wall Villages: A case study of three villages in Chicheng County
Source: PLoS One. 2024 Apr 18;19(4):e0298953. doi: 10.1371/journal.pone.0298953 (PMC11025826; doi:10.1371/journal.pone.0298953)
Supplement: S3 Table — (PDF) [file pone.0298953.s006.pdf]

S3 Table. Indices of the cultural landscape resilience of the Great Wall Villages.

Target	Criteria	Factor layer	Index layer	Weight	Score Standards
Evaluation of cultural landscape resilience of the Great Wall Villages A	Resistance B1 0.4934	Stability of ecological environmentC1 0.0828	Vegetation coverD1	0.0414	Direct use
			Landscape patch densityD2	0.0207	Direct use
			Topography D3	0.0207	Score1-5, according to the grade no, low, medium, high, very high suitability
		Integrity of defensive villagesC2 0.2072	Deterioration degree of the remainsD4	0.0714	1no remains, 2visible, 3serious damage, 4basic preserved, 5well preserved
			Longevity of historyD5	0.0225	1(1573-1620),2(1522-1566),3(1450-1521),4(1368-1453),5(1368)
			Military gradeD6	0.1133	Score1-5, according to grade Bao, Suo,Wei,Lu,Zhen citadel
		Preservation of spatial patternC3 0.1426	Site suitability D7	0.0240	1not suitable,3moderate,5suitable
			Integrity of village morphology D8	0.0550	1destruction,3partial change,5fully reserved
			Completeness of public facilitiesD9	0.0345	1incomplete,3moderate,5complete
			Road accessibility D10	0.0291	Score1-5, according to the no, low, medium, high, very high accessibility
		Stability of social structureC4 0.0608	Degree of folklore maintenanceD11	0.0300	Score1-5, according to the no, low, medium, high, very high maintenance
			The proportion of indigenous people in the total population D12	0.0189	Direct use
			The proportion of people leaving village D13	0.0119	Direct use

Recovery B2 0.1958	Diversity of landscape patterns C5 0.0264	Landscape diversity index D14	0.0132	Direct use
		Landscape dominance index D15	0.0132	Direct use
	Conservation and use of defensive buildingsC6 0.0843	Protection level of defensive buildingsD16	0.0327	Score1-5, according to the grade general, county, city, province, country
		Grade of villageD17	0.0143	Score1-5, according to the grade village,town,county,city
		Historic building renovation rate D18	0.0373	Score1-5, according to the no, low, medium, high, very high utilization rate
	Diversity of production spaceC7 0.0481	Spatial richness of village D19	0.0150	1have basic street system,3have historical buildings,5have street system and functional buildings
		Regional recognition of street landscape D20	0.0237	Score1-5, according to the no, low,medium, high,very high recognition
		Agricultural landscape types D21	0.0094	Score1-5, according to number of types1,2-4,4-6,6-8,8+
	Social cohesion of village C8 0.0370	Villagers' sense of belonging D22	0.0143	Score1-5, according to the no, low, medium, high, very high belonging
		Political participation D23	0.0164	Score1-5, according to the no, low, medium, high, very high participation
		Labor force ratio D24	0.0063	Direct use
Learning B3 0.3108	Historical and cultural inheritance C9 0.0526	Attractiveness folklore activities D25	0.0394	Score1-5, according to the no, low, medium, high, very high participation
		Richness of intangible culture D26	0.0132	Score1-5, according to no, less,medium, many,too many types
	Villager participation C10	Awareness of cultural heritage protection D27	0.0680	Score1-5, according to the no, low, medium, high, very high awareness

	0.1378	Heritage acceptance D28	0.0428	Score1-5, according to the no, low, medium, high, very high accaptance
		Proportion of educated population D29	0.0270	Direct use
		Relevant policy mechanisms D30	0.0374	1no supported policies, 3fewsupported policies,5 many supported policies
	Community learning C11 0.1204	Conservation, development planning D31	0.0594	1no planning,3 Planned but not implemented,5planning and implementation
		Extent of tourism development D32	0.0236	1 commercialisation,3moderate commercialisation,5no commercialisation
