# Supplementary material for: Cultural landscape resilience evaluation of Great Wall Villages: A case study of three villages in Chicheng County
Source: PLoS One. 2024 Apr 18;19(4):e0298953. doi: 10.1371/journal.pone.0298953 (PMC11025826; doi:10.1371/journal.pone.0298953)
Supplement: S4 Table — (PDF) [file pone.0298953.s007.pdf]

Questionnaire of Cultural Landscape Resilience Evaluation of Great Wall Villages

Hello! We are conducting a study on the resilience of the cultural landscape of the Great Wall Villages. Please complete this questionnaire according to your own feelings and experiences; the data is used for academic research and is filled out anonymously. Thank you for your support and cooperation in this survey.

Sex: ①male ②female
Age: ①Under 18 ②19-29 years old ③30-60 years old ④60 years and over
Your level of education: ①primary school ②junior high school ③senior high school ④technical secondary school ⑤junior college ⑥bachelor ⑦master and over

1. Do you think the terrain is flat and open enough to live in?
① very high suitability ② high suitability ③ medium suitability ④ low suitability ⑤ no suitability
2.How well do you think the defensive buildings have been preserved?
①well preserved ②basic preserved ③serious damage ④visible ⑤no remains
3.Do you think the location of the village blends in with the natural environment?
①not suitable ③moderat ⑤suitable
4. Do you think the public facilities in the village are complete for daily use?
①incomplete ③moderate ⑤complete
5. Do you think the current village pattern retains the historical settlement pattern?
①destruction ③partial change ⑤fully reserved
6. Do you think the village is easily accessible?
①no accessibility ②low accessibility ③medium accessibility ④high accessibility ⑤very high accessibility
7. Do you think that the streetscape in the village has a regional character?
①no accessibility ②low ③medium ④high ⑤very high
8. Do you think the local intangible culture is rich?
①no ②less ③medium ④many ⑤too many types
9. Do you participate in a lot of village folklore events?
①no ②low ③medium ④high participation ⑤very high participation
10. Do you have a sense of belonging to the village?
①no ②low ③medium ④high participation ⑤very high belonging
11. Would you actively participate in a campaign to protect the cultural heritage of the Great Wall?
①no ②low ③medium ④high participation ⑤very high participation
12. Have you paid attention to the information related to the development and preservation of the Great Wall?
①no ②low ③medium ④high focus ⑤very high focus

13. Do you know anything about cultural heritage?
①no ②low ③medium ④high realization ⑤very high realization
14. What do you think about the efficiency of village government management?
①no ②low ③medium ④high efficiency ⑤very high efficiency
15. Are you satisfied with status of development of the village now?
①no ②low ③medium ④high satisfaction ⑤very high satisfaction
16. What do you know about the history and culture of the military defense villages of the Great Wall?
①no ②low ③medium ④high realization ⑤very high realization
17. What kind of protection policies do you think the government should adopt?
①Encourage local governments and civic teams to organize relevant activities and increase publicity ②Encouragement and incentives for active participation of the population in conservation activities ③Establishment by the Government of a specialized agency for external fund-raising
18.In terms of publicity, what are your preferred ways to publicize the protection of cultural heritage?
①Documentary filming ②Organize activities ③Distribution of various materials such as brochures, posters, etc.
19. What do you think is the biggest problem facing the protection of cultural heritage today?
①Lack of effective protection mechanisms ②Conflict between foreign and modern cultures ③Lack of funds ④Aging of the inheritors
20. What do you think the government should do to protect historical and cultural landscapes?
①Government funding or external fund-raising for repairs ②Encourage active public participation in conservation actions ③Strengthening media outreach efforts ④others
21. Which of the following forces do you think are more important for the preservation of historic and cultural landscapes?
①government organization ②masses ③community organization ④other
22. Could you raise suggestions for the preservation or development of the Great Wall villages?
