# Supplementary material for: Cultural landscape resilience evaluation of Great Wall Villages: A case study of three villages in Chicheng County
Source: PLoS One. 2024 Apr 18;19(4):e0298953. doi: 10.1371/journal.pone.0298953 (PMC11025826; doi:10.1371/journal.pone.0298953)
Supplement: S5 Table — (PDF) [file pone.0298953.s008.pdf]

S5 Table. The resilience evaluation result of CLGWVs

Target	Criteria	Factor layer	Index layer	Weight	NYB	DSK	LMS
Evaluation of cultural landscape resilience of GWVs A	Resistance B1 0.4934	C1 0.0828	D1	0.0414	54.3%	37%	62%
			D2	0.0207	324.64	401.23	61.33
			D3	0.0207	3	4	5
		C2 0.2072	D4	0.0714	4	4	3
			D5	0.0225	2	4	4
			D6	0.1133	1	3	2
		C3 0.1426	D7	0.0240	4	5	3
			D8	0.0550	3	5	1
			D9	0.0345	1	3	4
			D10	0.0291	3	4	5
			D11	0.0300	3	3	3
		C4 0.0608	D12	0.0189	98.2%	97.4%	96%
			D13	0.0119	23.6%	38.5%	38.4%
	Recovery B2 0.1958	C5 0.0264	D14	0.0132	0.75	0.79	0.44
			D15	0.0132	1.33	1.27	2.25
		C6 0.0843	D16	0.0327	4	4	4
			D17	0.0143	1	2	2
			D18	0.0373	2	2	4
		C7 0.0481	D19	0.0150	1	2	3
			D20	0.0237	3	2	3
			D21	0.0094	2	2	4
		C8 0.0370	D22	0.0143	4	3	2
			D23	0.0164	3	3	4
			D24	0.0063	67%	71.47%	70%
	Learning B3 0.3108	C9 0.0526	D25	0.0394	3	3	4
			D26	0.0132	1	2	3
		C10 0.1378	D27	0.0680	3	3	4
			D28	0.0428	2	3	3
			D29	0.0270	1	2	3
			D30	0.0374	2	5	3
		C11 0.1204	D31	0.0594	2	4	3
			D32	0.0236	3	4	3

NYB refers to Ningyuanbao Village, DSK refers to Dushikou Village, LMS refers to Longmensuo Village.
