# Supplementary material for: Cultural landscape resilience evaluation of Great Wall Villages: A case study of three villages in Chicheng County
Source: PLoS One. 2024 Apr 18;19(4):e0298953. doi: 10.1371/journal.pone.0298953 (PMC11025826; doi:10.1371/journal.pone.0298953)
Supplement: S2 Fig — (A) Geographical location of Dushikou Village. The base map was from USGS EROS: http://eros.usgs.gov/#. Yellow line reprsents fort, blue line represents river, orange line represents the Great Wall. (B) Current spatial layout of Dushikou Village. The figure was made by Weiya Zhang. The thinnest light gray line represents streets, pink area represents original site area, blue-gray line represents external transport, dark gray line represents national roads, the light blue areas represent rivers. (C) The Great Wall in Dushikou Village. (D) The southern wall of Dushikou Fortress. (E) Beacon tower. (F) Pailou at western gate of Dushikou Village. (G) Rock called Dushi. The photos of (C)-(G) were provided by Xiaodong Ming. (PDF) [file pone.0298953.s002.pdf]

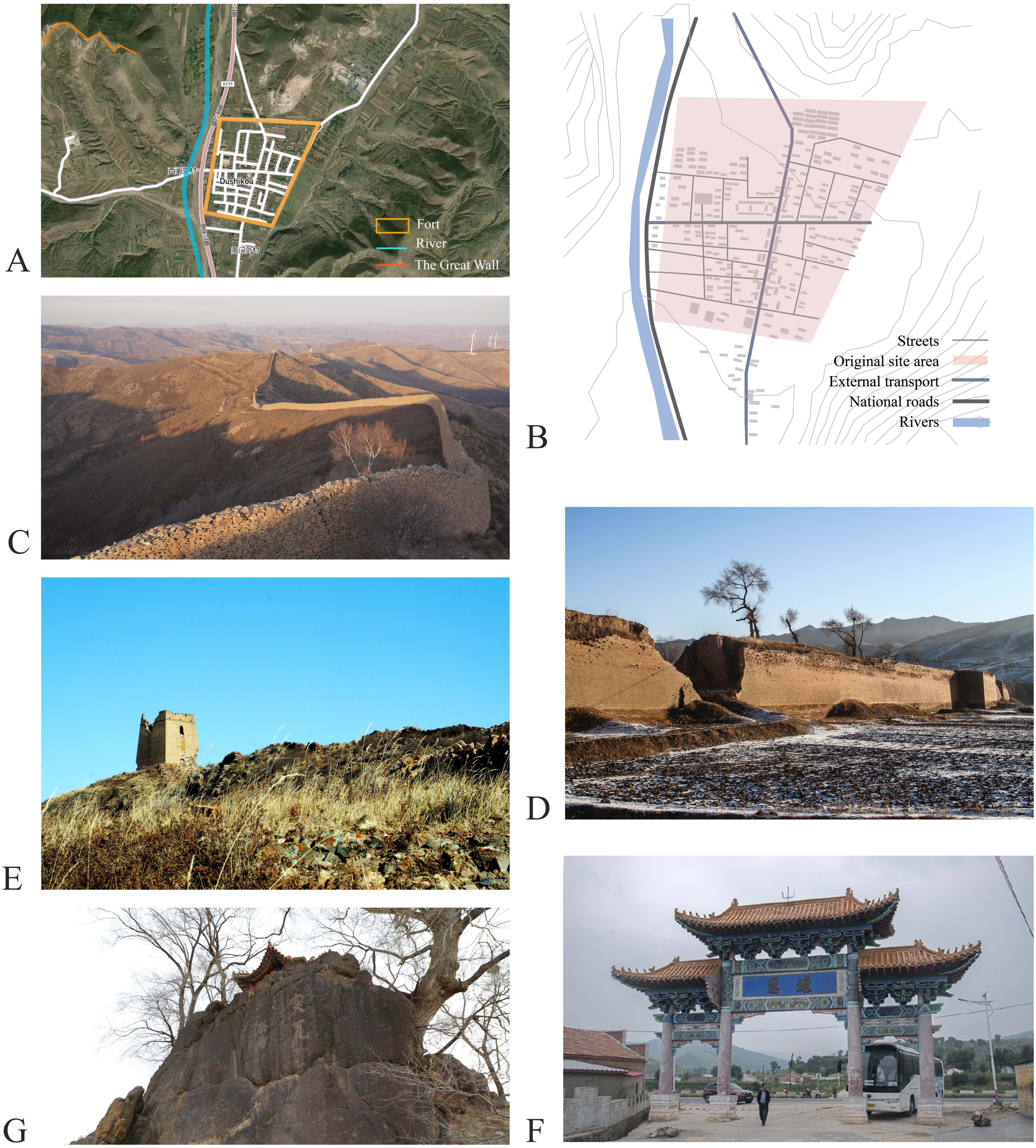
A
Dushikou
西门外村
南门外村
Fort
River
The Great Wall
B
Streets
Original site area
External transport
National roads
Rivers
C
D
E
F
G
